# Occurrence of *Prototheca* Microalgae in Aquatic Ecosystems with a Description of Three New Species, Prototheca fontanea, Prototheca lentecrescens, and Prototheca vistulensis

**DOI:** 10.1128/aem.01092-22

**Published:** 2022-10-27

**Authors:** Tomasz Jagielski, Mateusz Iskra, Zofia Bakuła, Joanna Rudna, Katarzyna Roeske, Julita Nowakowska, Jacek Bielecki, Henryk Krukowski

**Affiliations:** a Department of Medical Microbiology, Institute of Microbiology, Faculty of Biology, University of Warsaw, Warsaw, Poland; b Laboratory of Electron & Confocal Microscopy, Faculty of Biology, University of Warsaw, Warsaw, Poland; c Department of Animal and Environmental Hygiene, University of Life Sciences in Lublin, Lublin, Poland; Royal Netherlands Institute for Sea Research

**Keywords:** *Prototheca* sp., algae, aquatic ecosystem, environment, waterbody

## Abstract

*Prototheca* species are unicellular, nonphotosynthetic, saprophytic, and occasionally pathogenic, microalgae, with an extensive environmental reservoir. This study explores, for the first time, the occurrence of *Prototheca* in aquatic ecosystems by using a molecular profiling approach. A total of 362 samples were collected from 80 natural and artificial waterbodies at 88 sampling sites in 26 localities across Poland during a 1.5-year period. The overall isolation rate of *Prototheca* from water environments was 14.1%. *Prototheca* were most prevalent in rivers of urbanized areas, indicating that the algae are primarily adapted to lotic ecosystems with a high input of organic matter. Interestingly, it is not the amount of organic matter *per se* but its quality that seems to shape the habitat potential of the protothecae. The two most frequently isolated species were P. wickerhamii and P. pringsheimii, representing a third and a fourth of the strains, respectively. Additionally, three novel species were described, namely, P. fontanea, P. lentecrescens, and P. vistulensis. The high species diversity of the genus *Prototheca* may reflect the complexity of water ecosystems along with ecological and functional adaptations of the algae to such environments. For further investigations, the study provides a revised scheme for identification of all 18 *Prototheca* species currently recognized.

**IMPORTANCE** The study investigates the occurrence of very rare and poorly studied microalgae of the genus *Prototheca*, potentially pathogenic to humans and animals, in different water environments. Given the potential hazard to human and animal health from exposure to water-inhabiting protothecae, the prevalence of the algae in aquatic habitats deserves an insightful examination. The study is the first since the 1980s to explore the aquatic habitat of *Prototheca* spp. and the first ever performed to do this by molecular methods. Although the *Prototheca* isolation rate was low, a high species diversity was observed. The algae appear to represent allochthonous microflora, brought into waterbodies from various anthropogenic sources. Large rivers of urbanized areas were the most *Prototheca*-abundant. The study provides a description of three new *Prototheca* species, namely, *P. fontanea*, *P. lentecrescens*, and *P. vistulensis*. The study also delivers a new identification scheme for all *Prototheca* species currently recognized.

## INTRODUCTION

The *Prototheca* genus (Trebouxiophyceae) comprises unicellular, nonphotosynthetic, saprophytic microalgae associated with rare but severe infections representing a potential zoonotic risk (1). *Prototheca* are the only algae which have repeatedly been reported to infect vertebrates, including humans, causing a variety of pathologies, collectively referred to as protothecosis. In animals, the disease most commonly affects dairy cattle, resulting in clinical or subclinical mastitis, while in humans the predominant manifestations are linked to cutaneous, articular, and systemic involvement (2–4). The taxonomy of the *Prototheca* genus has long been contentious and frequently revised. Recent studies based on the phylogenetic analysis of the apocytochrome *B*-coding sequence data have led to the establishment of a new taxonomic classification system of the *Prototheca* algae, installing within the genus a total of 14 species (5). Of these, six have been recognized as opportunistic pathogens, while the remainder have so far been cultured from environmental sources only.

*Prototheca* are ubiquitous microorganisms, with a strong predilection for areas of high organic matter and moisture content. Over the years, many ecological niches have been identified for *Prototheca* spp. The algae were primarily recovered from plant material, such as slime flux of elm, lime, mulberry, and oak trees (6–9), bark of a cherry tree (10), fruit coat of loquat (11), cut stems of banana plants (12), potato epidermis (13), plant debris (3), and lichens (14). However, *Prototheca* algae have also been isolated from terrestrial sources, such as soil, mud, and stream sediments (3, 9, 15, 16). Much of the currently known *Prototheca* environmental reservoir has been described on dairy farms, since a number of species, most notably P. bovis and P. blaschkeae, are the causative agents of bovine mastitis (3). Several studies have shown cow barn surroundings (bedding material, manure, feeders, water troughs, and excreta) as important sources of algal contamination (3, 17–20). Likewise, the milking parlor, teat cup liners, pipelines, milk cooling bulk tanks, and other milking utensils have often been contaminated with *Prototheca* spp. (3, 17–21). Perturbingly, their occurrence in the dairy herd environment is persistent and hardly eliminable by the common sanitation and disinfecting practices (1, 3).

This persistence is thought to be conveyed by the presence of sporopollenin, a highly durable, cross-linked, carotenoid polymer, in the algal cell walls, which makes them refractory to desiccation, enzymatic digestion, mechanical damage, and other physical and chemical treatments. It is plausibly due to sporopollenin that the *Prototheca* species withstand standard chlorination in sewage treatment systems or resist high temperatures, including those used for milk pasteurization (22–24). The ability of the protothecae to survive hostile and harsh environmental conditions increases the risk of their transmission to animals or humans.

*Prototheca* spp. strains have been isolated from both natural water sources, including streams, rivers, lakes, and artificial aquatic reservoirs, such as municipal tap water, cattle drinking troughs, irrigation canals, fishponds, aquarium tanks, wastewater effluent, and water treatment facilities (3, 9, 25–27). The algae tolerate high salinities and are found more often in turbid than clear water, which may relate to their cell surface hydrophobicity, at least in noncapsulated species (9). Despite their environmental ubiquity, the *Prototheca* algae are considered to be transient inhabitants of water environments, which they enter with rainfall washing them out from external sources (e.g., slime flux, soil, sludge) (9). Still, wastewater is one of the primary sources of *Prototheca* algae, with P. wickerhamii being the most frequently isolated species (9, 25, 27).

Aquatic and terrestrial environments contaminated with the *Prototheca* algae may provide a source of infection for humans and animals through either direct contact or traumatic inoculation (1, 2). The vast number of documented cases of protothecosis with a clearly or potentially defined portal of infection have pointed to *Prototheca*-infested water as a vehicle of disease transmission (2, 28). Considering the potential hazard to human and animal health from exposure to water-inhabiting protothecae, along with their reported long-term survival under different water regimes, the prevalence of the algae in aquatic habitats deserves an insightful examination. Since the mid-1980s, no studies have been undertaken to cross-sectionally examine the occurrence of the *Prototheca* algae in natural aquatic environments. Nor have the relationships between the prevalence of a given *Prototheca* species and the features of the water environment been explored. The aim of the study was therefore to investigate the occurrence of protothecae in a wide range of freshwater ecosystems in Poland and demonstrate the species diversity of these algae by using a molecular taxonomic profiling approach.

## RESULTS

### Prevalence of *Prototheca* algae in water samples.

Of 362 samples collected, 299 (82.6%) were liquid and 63 (17.4%) were solid samples. Most samples originated from 62 natural water environments (277/362; 76.5%). The remaining samples were collected from 18 artificial reservoirs (85/362; 23.5%) (see Table S1 in the supplemental material).

A total of 51 (14.1%) samples collected from 15 environmental sites in 10 localities of all provinces except Podlasie yielded growth of *Prototheca* spp. (Table 1; Table S1). The strains cultured were of both aqueous and sedimentary origin (37 versus 14 strains) (Table S1). They were recovered most frequently from rivers and streams (40/114 samples or 35.1%; 30 aqueous and 10 solid samples), with only the Vistula River accounting for 23 strains (23/51 or 45.1%). A total of 17 (33.3%) *Prototheca* strains were isolated from minor rivers (or streams) in Subcarpathia (Wielopolka, 7), Łódź (Strawa, 3), Mazovia (Służewiecki Stream, 2; Narew, 2; Omulew, 1), and Lesser Poland (Foluszowy Stream, 1; Bystra, 1). Of these, the most *Prototheca*-abundant was the urban Wielopolka River corridor in Ropczyce, from where 6 out of 9 (66.6%) samples were *Prototheca* positive (Table S1).

**TABLE 1:** Sampling localities, types of collected samples and number of *Prototheca* spp. isolates cultured^*a*^

		Type of sample		Type of reservoir			
Voivodeship	Locality	Liquid	Solid	Rivers/streams	Lakes/ponds/wetlands	Artificial	No. of samples
Kuyavia-Pomerania	Ciechocinek	8 (1)	2	10 (1)			10 (1; 10%)
		**8** (1; 12.5%)	**2**	**10** (1; 10%)			**10**
Lesser Poland	Cracow	11 (3)	2	3 (3)		10	13 (3; 23.1%)
	Zakopane	8 (2)	2	10 (2)			10 (2; 20%)
		**19** (5; 26.3%)	**4**	**13** (5; 38.5%)		**10**	**23** (5; 21.7%)
Łódź	Piotrków	6 (2)	2 (1)	8 (3)			8 (3; 37.5%)
	Trybunalski	**6** (2; 33.3%)	**2** (1; 50%)	**8** (3; 37.5%)			**8**
Mazovia	Cegłów	1				1	1 (0; 0%)
	Drężewo	1 (1)		1 (1)			1 (1; 100%)
	Izdebno Kościelne	2				2	2 (0; 0%)
	Izdebno Nowe	1				1	1 (0; 0%)
	Leszno	10			10		10 (0; 0%)
	Modlin	2 (2)		2 (2)			2 (2; 100%)
	Ostrołęka	2		1		1	2 (0; 0%)
	Skuszew	1			1		1 (0; 0%)
	Warsaw	191 (22)	42 (8)	58 (21)	117 (6)	58 (3)	233 (30; 12.9%)
	Wyszków	1		1			1 (0; 0%)
	Zabłotnia	1				1	1 (0; 0%)
	Zielonka	10		2	8		10 (0; 0%)
		**223** (25; 11.2%)	**42** (8; 19.1%)	**65** (24; 36.9%)	**136** (6; 4.4.%)	**64** (3; 4.8%)	**265** (33; 12.5%)
Podlasie	Suwałki	4	1	2		3	5 (0; 0%)
		**4**	**1**	**2**		**3**	**5**
Subcarpathia	Borek mały	2			2		2 (0; 0%)
	Kamionka	5	1			6	6 (0; 0%)
	Kozodrza	5	2 (1)	7 (1)			7 (1; 14.3%)
	Ostrów	7	2		9		9 (0; 0%)
	Ropczyce	7 (4)	2 (2)	9 (6)			9 (6; 66.7%)
	Zdżary	2				2	2 (0; 0%)
		**28** (4; 14.3%)	**7** (3; 42.9%)	**16** (7; 43.8%)	**11**	**8**	**35** (7; 20%)
Warmia-Masuria	Mikołajki	7	4 (2)		11 (2)		11 (2; 18.2%)
	Ostróda	1			1		1 (0; 0%)
	Pilchy	3	1		4		4 (0; 0%)
		**11**	**5** (2; 40%)		**16** (2; 12.5%)		**16** (2; 12.5%)
Total		**299** (37; 12.4%)	**63** (14; 22.2%)	**114** (40; 35.1%)	**163** (8; 4.9%)	**85** (3; 3.5%)	**362** (51/362; 14.1%)
		362		362			

a Numbers of *Prototheca* spp. isolates are given in parentheses. Percentages were calculated with reference to all samples of a given type. Voivodeship and country totals are in bold type.

The prevalence of the algae in stagnant waters of lakes and ponds (8/163 or 4.9%; 4 water and 4 solid samples) was 7-fold lower than in flowing waters. The *Prototheca* isolation from artificial reservoirs was the least successful (3/85 or 3.5%). Here, the only three strains were isolated from the Żerański canal (2 strains) and a municipal fountain in Warsaw (one strain), both located in central Poland (Mazovia).

The overall isolation rate of *Prototheca* spp. from water environments was 14.1% (51/362), with the within-site prevalence ranging from 0% (73 sites in 19 localities) to 100% (4 sites in 3 localities) (Table S1). At the voivodeship level, the highest proportion of *Prototheca*-positive samples was observed in Łódź (3/8 or 37.5%), followed by Lesser Poland (5/23 or 21.7%), Subcarpathia (7/35 or 20%), Warmia-Masuria (2/16 or 12.5%), Mazovia (33/265 or 12.5%), and Kuyavia-Pomerania (1/10 or 10%). No *Prototheca* spp. were detected only in the utmost southeast of Poland (Podlasie).

### *Prototheca* spp. identification and phylogeny.

**FIG 1:**
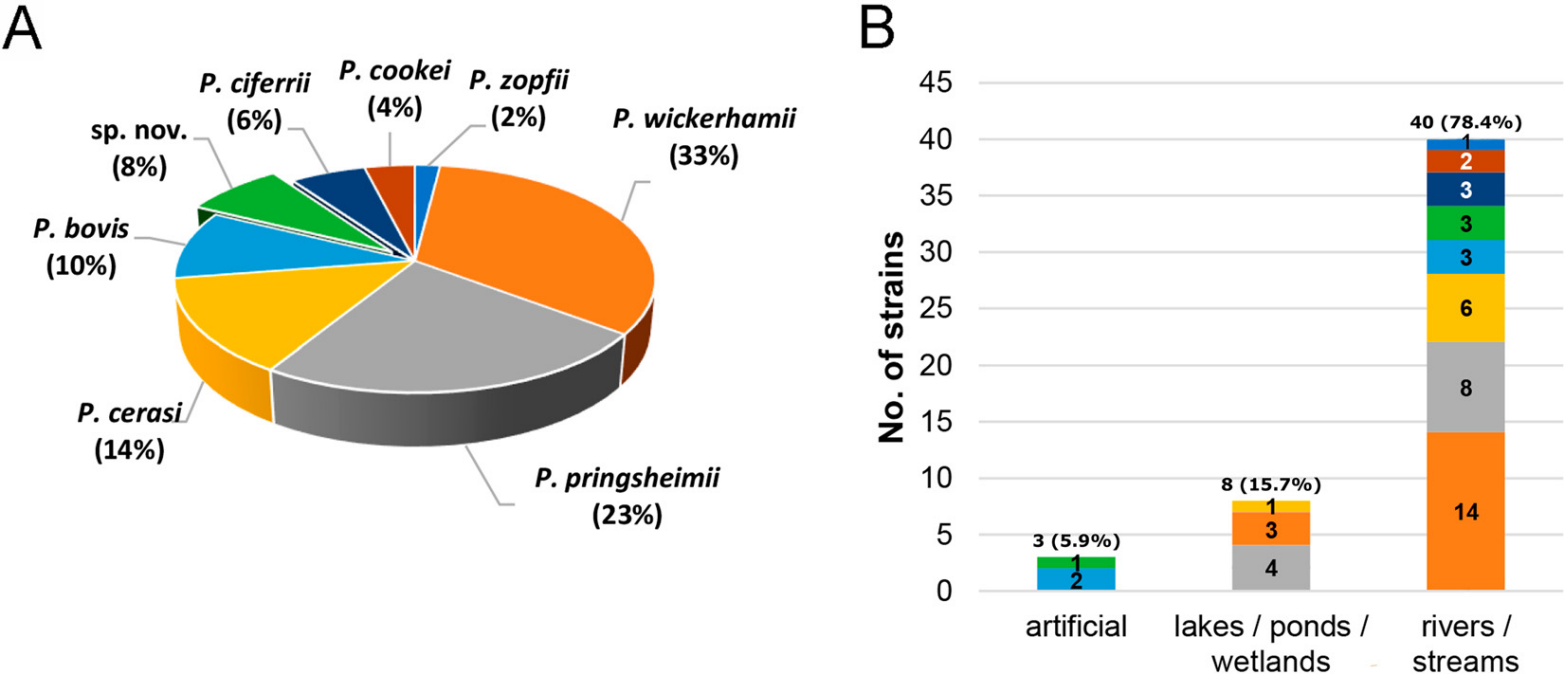
(A and B) Species profiling of *Prototheca* strains isolated from all sampling sites (A) and by type of reservoir (B).

All 51 *Prototheca* isolates cultured were subjected to partial *CYTB*-based PCR-restriction fragment length polymorphism (RFLP) profiling, and 47 (92%) were thereby successfully identified at the species level. Of these, the majority were identified as *P. wickerhamii* (17/51, 33%), followed by P. pringsheimii (12/51, 23%), P. cerasi (7/51, 14%), *P. bovis* (5/51; 10%), P. ciferrii (3/51; 6%), P. cookei (2/51; 4%), and P. zopfii (1/51; 2%) (Fig. 1). The majority of the environmental isolates collected from rivers/streams were *P. wickerhamii* (14/40; 35%), followed by *P. pringsheimii* (8/40; 20%), *P. cerasi* (6/40; 15%), *P. bovis* (3/40; 7.5%), *P. ciferrii* (3/40; 7.5%), *P. cookei* (2/40; 5%), and *P. zopfii* (1/40; 2.5%). Among eight isolates collected from natural stagnant waters (lakes, ponds, wetlands), four isolates were *P. pringsheimii* (4/8; 50%), three isolates were *P. wickerhamii* (3/8; 37.5%), and one isolate was *P. cerasi* (1/8 12.5%). Of three isolates collected from artificial reservoirs, two were *P. bovis* (2/3; 66.7%) (Fig. 1).

**FIG 2:**
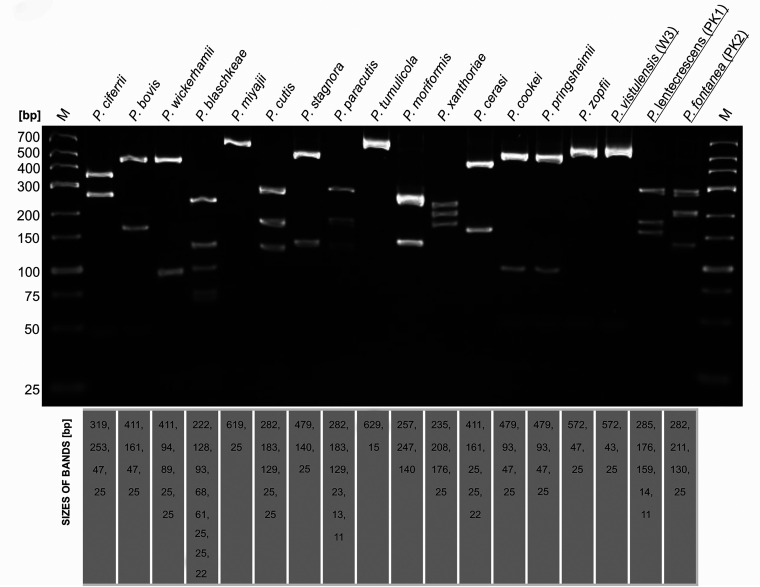
PCR-RFLP patterns of *Prototheca* spp., including three novel species (underlined), as achieved by amplification of the partial *CYTB* gene sequences and TaiI/RsaI double digestion of the amplicons. M, size marker (GeneRuler low range, Thermo Scientific, Waltham, MA, USA). The bottom panel provides sizes of the restriction fragments produced for each species.

**FIG 3:**
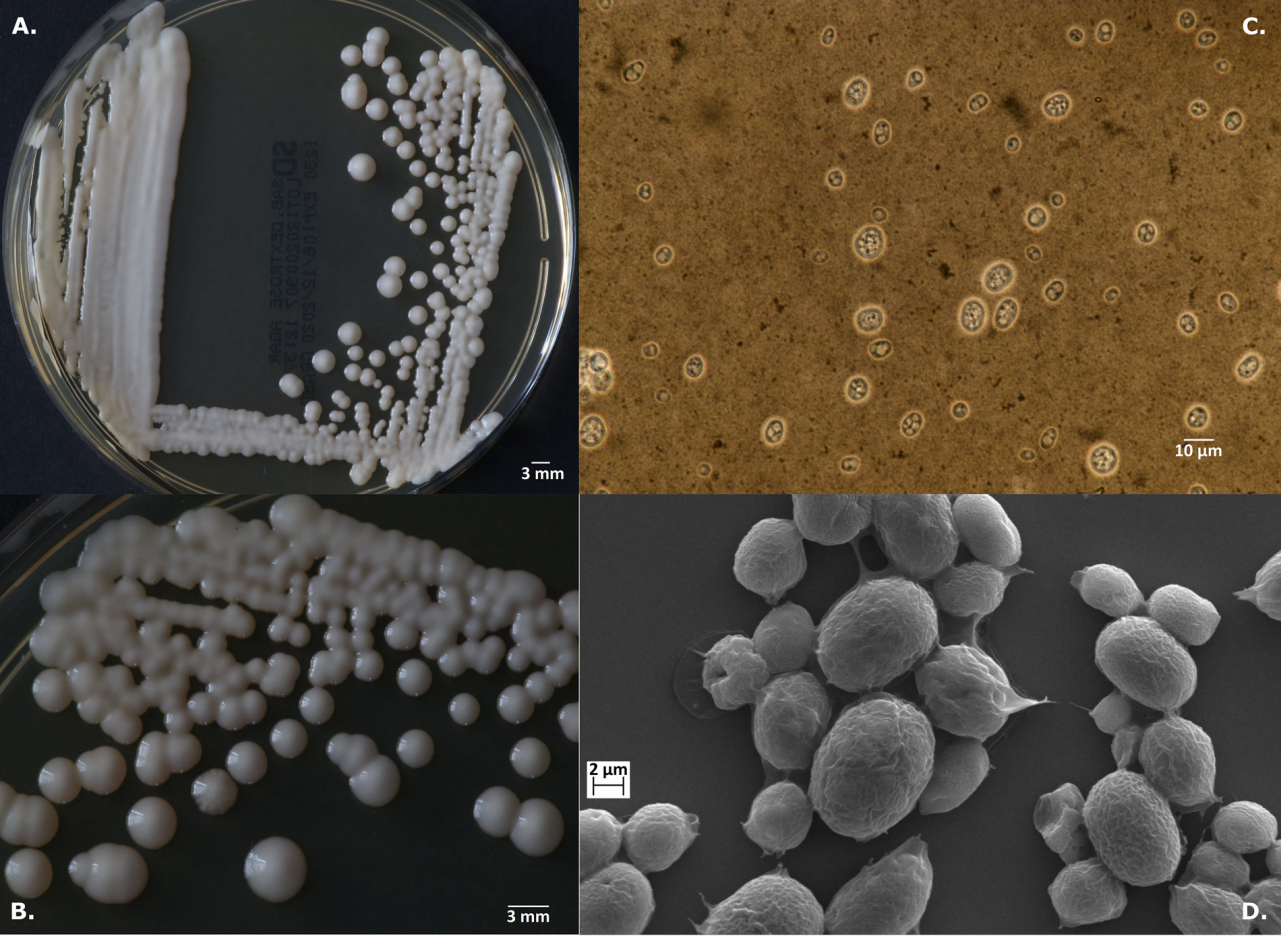
*Prototheca vistulensis* sp. nov. strain W3 type strain (T). (A and B) Colonies on SDA medium after 72 h at 25°C. (C and D) Details of cell morphology, as seen using optical microscopy (nigrosine stain, ×1,000)(C) and SEM (×6,000) (D).

Of the four isolates for which PCR-RFLP profiling was inconclusive (PK1, PK2, PK6, W3), three (PK1, PK2, PK6) exhibited unusual banding patterns not conforming to those reported for any known *Prototheca* species. The PCR-RFLP profile of one strain (W3) was nearly identical to that of *P. zopfii* (Fig. 2). However, the strain differed from the latter in the glistening and slimy appearance of its colonies and the presence of the capsule (Fig. 3).

The four strains suspected of representing potentially new *Prototheca* species were subjected to further molecular analysis by means of sequencing of the partial *CYTB* gene and the small subunit (SSU), large subunit (LSU), and internal transcribed spacer 1 (ITS1)/2 loci. However, since PK1 and PK6 strains were isolated from the same sampling site, yielded the same PCR-RFLP restriction profile, and showed *CYTB* sequence homology of 99.8%, they were considered conspecific, and only PK1, as a representative, was sequenced in rDNA regions.

**FIG 4:**
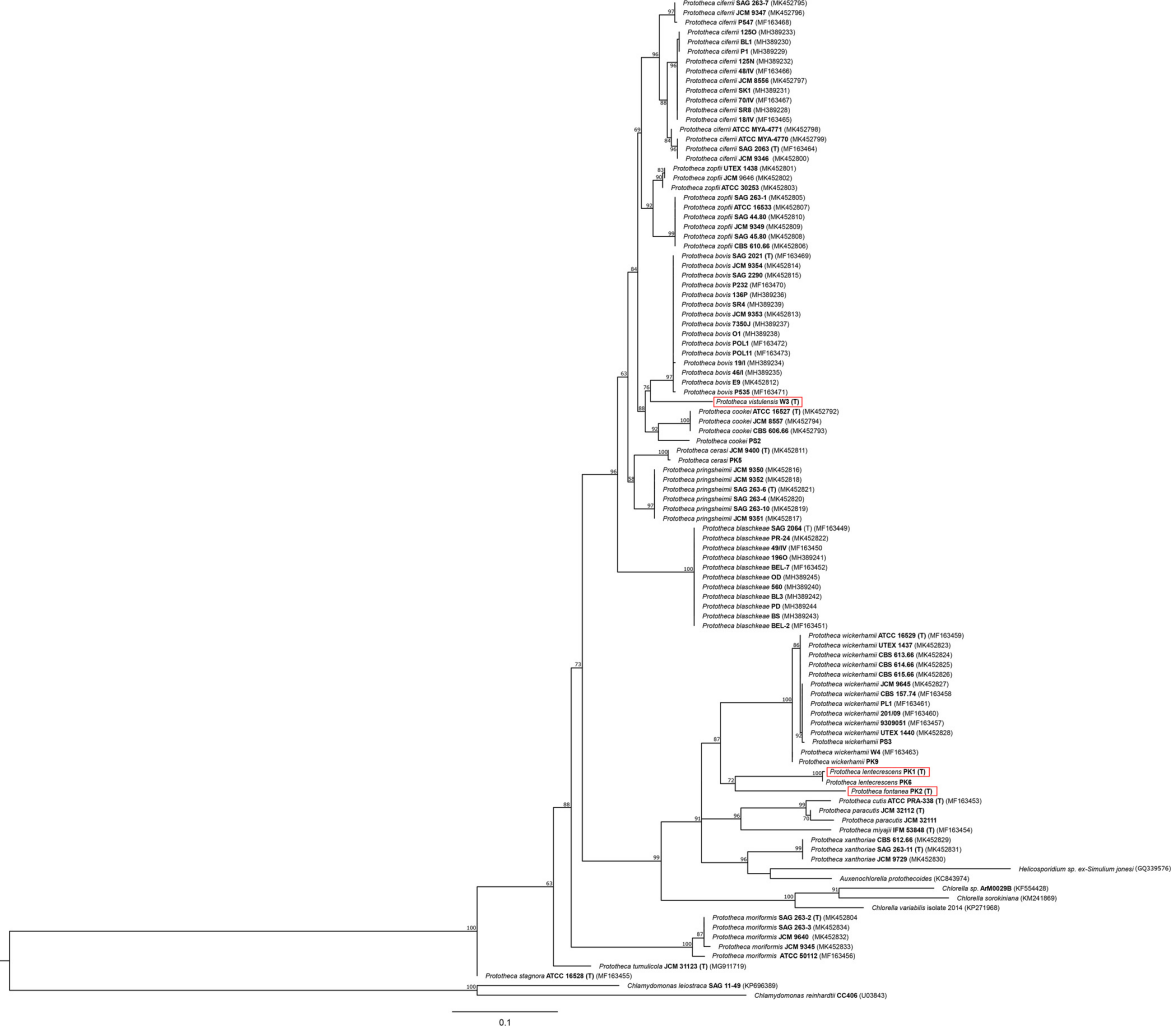
Phylogenetic tree constructed by maximum likelihood (ML) analysis based on 103 partial *CYTB* gene sequences of *Prototheca* spp. (96 sequences) and 4 related taxa (7 sequences). Numbers at the nodes are ML bootstrap values (bs) above 50%. The phylogram was rooted to *Chlamydomonas leiostraca* (SAG 11-49) and *Chlamydomonas reinhardtii* (CC 406). All strains are presented under their original species (varietal) names with corresponding numbering (in bold), as accessed in culture collections. Type strains are indicated with a bracketed T, and for three newly proposed species, their names and strain numbers are red-boxed. The scale bar indicates 1 substitution per 10 nucleotide positions.

For partial *CYTB* gene sequences (size 598 to 668 bp) a BLAST search was performed on the Prototheca-ID and GenBank databases. Upon alignment with 92 protothecal sequences and 7 equivalent sequences from related genera, a phylogram was constructed to assess phylogenetic relationships (Fig. 4). *CYTB* sequences of PK1/6, PK2, and W3 strains showed less than 90.7% similarity to each other and less than 95.4% to any other *Prototheca* species, proving their separate species status. PK1 and PK6 grouped together in a distinct cluster (P. lentecrescens), alongside strain PK2 (*P. fontanea*), with 90.6% and 90.7% sequence identity, respectively. This cluster formed a sister group to the *P. wickerhamii* clade, supported by a relatively high bootstrap value (87%). Interestingly, it was P. xanthoriae, not strain PK1 or PK6, that shared the highest *CYTB* sequence similarity (91.8%) with strain PK2.

The W3 strain nested within a clade formed with *P. bovis* and in a larger clade, which also included *P. cookei*. Strain W3 (P. vistulensis) clustered with the two species at 95.4% and 94.6% sequence similarity, respectively, and with all the remaining *Prototheca* species at 84.5 to 94.7% (Table S3).

Phylogenetic trees based on rDNA (LSU, SSU, and ITS) sequences accorded well with that inferred from the *CYTB* gene sequences, clearly separating strains PK1/6 (*P. lentecrescens*), PK2 (*P. fontanea*), and W3 (*P. vistulensis*) from other *Prototheca* spp. On each of the rDNA trees, these strains formed solitary branches, supporting their phylotaxonomic distinctiveness. Furthermore, as far as the strains in question are concerned, topologies of all four phylograms were highly congruent (Fig. S1 to S3). Based on the results of molecular analysis, three new species are proposed and their descriptions given below.

**FIG 5:**
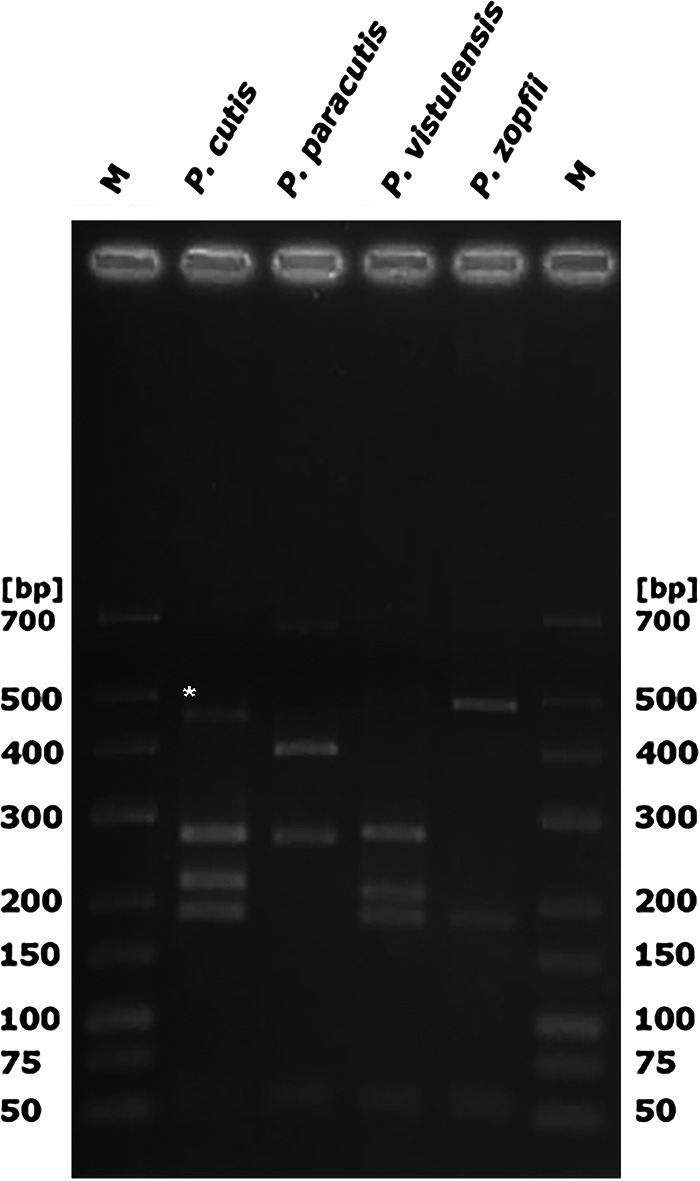
Digestion of partial *CYTB* amplicons of *P. vistulensis* and *P. zopfii* with VspI and of *P. cutis* and *P. paracutis* with BfaI; M, size marker (GeneRuler low range, Thermo Scientific, Waltham, MA, USA). Note that an unspecific band (ca. 480 bp) was occasionally present for *P. cutis* (*).

**FIG 6:**
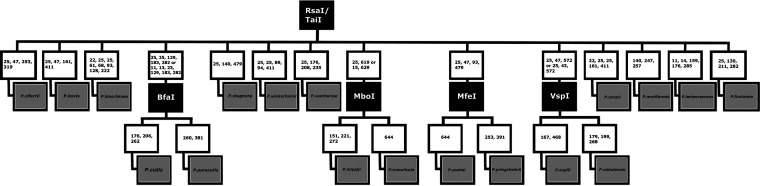
A modified algorithm for *Prototheca* spp. differentiation based on PCR-RFLP analysis of the partial *CYTB* gene. Restriction enzymes used and resulting restriction fragment lengths are boxed in black and white, respectively. Some strains of *P. ciferrii* produce a pattern characteristic for *P. bovis*.

**Identification of new species by PCR-RFLP analysis.** To develop PCR-RFLP assays allowing the identification of three newly described *Prototheca* species, the *CYTB* gene sequences of the new *Prototheca* sp. type strains were subjected to *in silico* digestions, with either a combination of RsaI and TaiI enzymes or extra new enzymes, so that species-specific patterns could be produced. All computer-simulated restrictions were carried out with Clone Manager software v9.0 (Sci-Ed Software, Denver, CO, USA). The *in silico* patterns were validated by *in vitro* restriction digestions: a double RsaI/TaiI digestion to produce unique patterns for *P. fontanea* and *P. lentecrescens* (Fig. 2) and a separate digestion with VspI to distinguish between *P. vistulensis* and *P. zopfii*. (Fig. 5). In addition, a newly designed PCR-RFLP assay with BfaI allowing for discrimination between P. cutis and P. paracutis was validated (Fig. 4, 5, and 6). The VspI and BfaI reactions were performed with FastDigest enzymes (Thermo Fisher Scientific, Waltham, MA, USA) with their mixtures containing 1× FastDigest buffer, 15 μL of PCR product, and 1 μL of each enzyme in a final volume of 30 μL. Digestions were performed at 37°C for 10 min. The restriction products were separated on 4% (wt/vol) agarose–Tris-borate-EDTA (TBE) gels for 90 min and visualized by ethidium bromide (EtBr) staining. Analysis of the electropherograms was done with the UVP BioDoc-IT imaging system (Analityk Jena, Jena, Germany).

### Description of new species.

***Prototheca vistulensis* Jagielski et Iskra sp. nov. (i) Description.** Sporangia are globose to ellipsoidal, measuring on average 9.9 by 9.3 μm; sporangiospores are globose or angular, measuring on average 4.1 by 3.4 μm; capsule is present; after a 72-h incubation on Sabouraud dextrose agar at 25°C, colonies are creamy-white, circular, raised, glistening, and slimy, with smooth surface and margins, of butyrous consistency, with up to 5 mm in diameter (Fig. 3); grows well at 25°C, 30°C, and at 35°C; assimilates glucose, glycerol, galactose, and trehalose (Table 2).

**TABLE 2:** Phenotypic characteristics of the novel *Prototheca* species with GenBank accession numbers for *CYTB* and rDNA markers

Species	Strain	Cell size (μm)^*a*^	Capsule	Growth at^*b*^:			Carbohydrate substrate^*c*^:					GenBank accession no.			
				25°C	30°C	35°C	Glu	Gly	Gal	Tre	Lat*	*CYTB*	SSU	LSU	ITS
*P. lentecrescens*	PK1 (T)	2.9–16.3 by 2.7–15.3	–	+/–	+/–	–	+	–	+	+/–	–	MW701399.1	MZ198751	OK236514.1	OK236512.1
*P. fontanea*	PK2 (T)	3.3–13.3 by 3.1–12.8	–	+	+	+/–	+	+	+	+	–	OK169375.1	MZ198752.1	OK236513.1	OK236511.1
*P. vistulensis*	W3 (T)	2.5–5.1 by 2.4–4.0	+	+	+	+	+	+	+	+	–	MN854981.1	MZ191909	MN846676.1	MN846677.1

a Dimensions of cells regardless of developmental stage are given as length by width.

b Growth on Sabouraud dextrose agar (SDA) medium after 72 h.

c All tests were done with the API ID32C system (bioMérieux, Marcy-l’Étoile, France), according to the manufacturer’s instructions; Glu, d-glucose; Gly, glycerol; Gal, d-galactose; Tre, d-trehalose. Lat*, lactic acid and others: Act, cycloheximide (actidion); Lac, d-lactose; Raf, d-rafinose; Mal, d-maltose; Sac, d-saccharose; 2KG, potassium 2-ketogluconate; Mdg, methyl-αd-glucopyranoside; Man, d-Mannitol; Cel, d-cellobiose; Ino, inositol; Sor, d-sorbitol; Xyl, d-xylose; Rib, d-ribose; Nag, *N*-acetyl-glucosamine; Rha, l-rhamnose; Ple, palatinose; Ery, erythritol; Mel, d-melbiose; Grt, sodium glucuronate; Mlz, d-melezitose; Gnt, potassium gluconate; Lvt, levulinic acid; Ara, l-arabinose; Sbe, l-sorbose; Gln, glucosamine; Esc, esculin. Growth: +, positive; +/–, weak; –, negative.

**(ii) Holotype.** Resin-embedded specimen of strain W3, deposited at the Herbarium of the Faculty of Biology at the University of Warsaw (Herbarium WA), Warsaw, Poland (type specimen no. WA0000125950).

**(iii) Type locality.** Water from the Vistula River. Cracow, Lesser Poland, Poland.

**(iv) Etymology.** The epithet *vistulensis* (vi.stu.len’sis. L. fem. n. *Vistula*, a river in Poland, L. fem. suffix -ensis indicating provenance, N.L. fem. nom. adj. *vistulensis*), isolated from the Vistula River in Poland.

**FIG 7:**
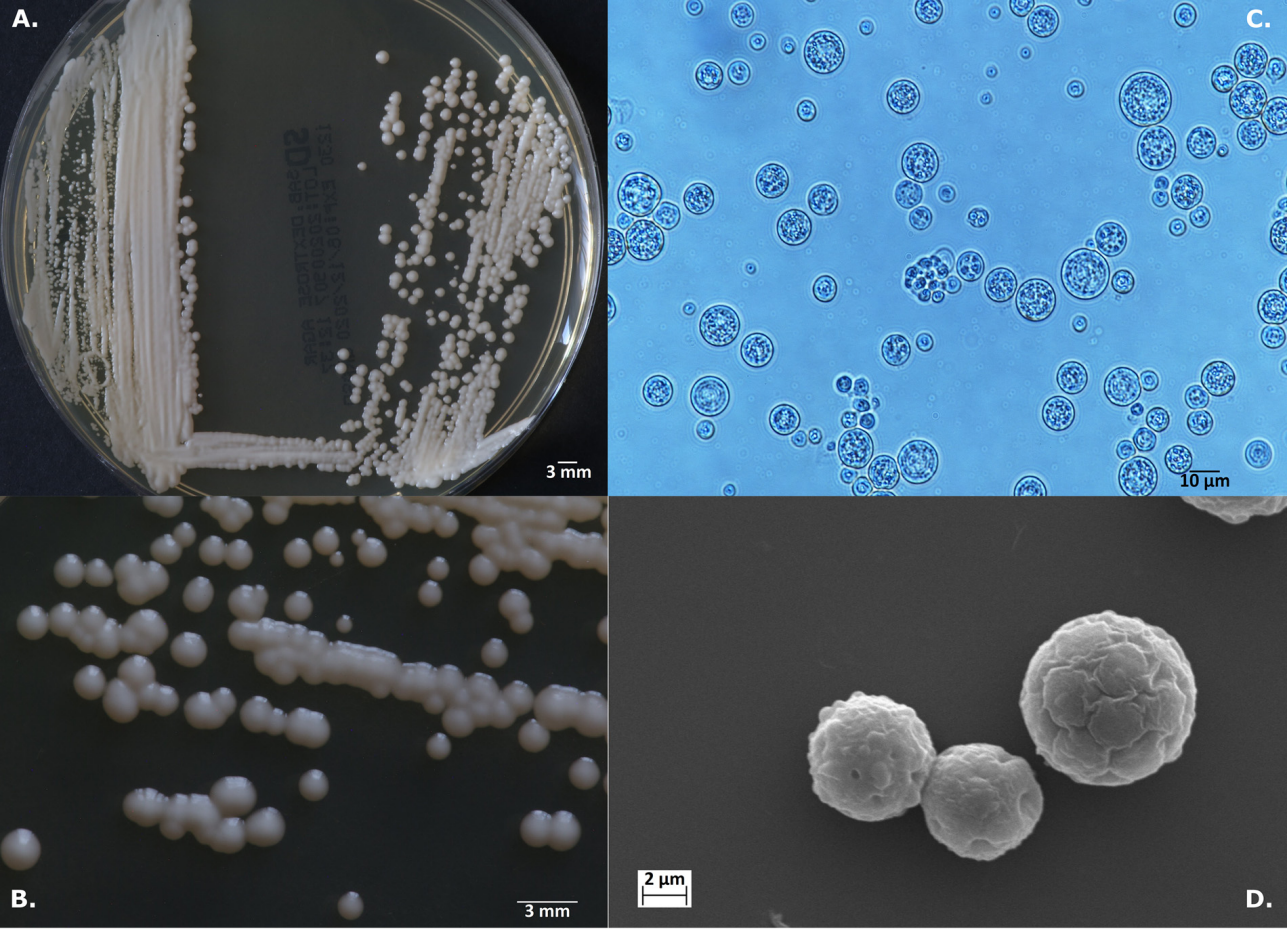
*Prototheca lentecrescens* sp. nov. strain PK1 type strain (T). (A and B) Colonies on SDA medium after 168 h at 25°C. (C and D) Details of cell morphology, as seen using optical microscopy (unstained, ×1,000) (C) and SEM (×6,000) (D).

***Prototheca lentecrescens* Jagielski et Iskra sp. nov. (i) Description.** Sporangia and sporangiospores are globose, measuring on average 13.9 by 13.7 μm and 9.8 by 9.5 μm, respectively; capsule is absent; after a 144-h incubation on Sabouraud dextrose agar at 25°C, colonies are creamy-white, circular, raised, glistening, with smooth surface and margins, of butyrous consistency, with up to 3 mm in diameter (Fig. 7); grows at 25°C and 30°C, but not at 35°C; assimilates glucose, galactose, and trehalose; glycerol is not assimilated (Table 2).

**(ii) Holotype.** Resin-embedded specimen of strain PK1, deposited at the Herbarium of the Faculty of Biology at the University of Warsaw (Herbarium WA), Warsaw, Poland (type specimen no. WA0000125952).

**(iii) Type locality.** Water from the Vistula riverbank. Warsaw, Mazovia, Poland.

**(iv) Etymology.** The epithet *lentecrescens* (len.te.cre’scens. L. adv. *lente*, slowly, L. v. *crescere*, grow, N.L. nom. fem. part. adj. *lentecrescens*), slowly growing (in culture).

**FIG 8:**
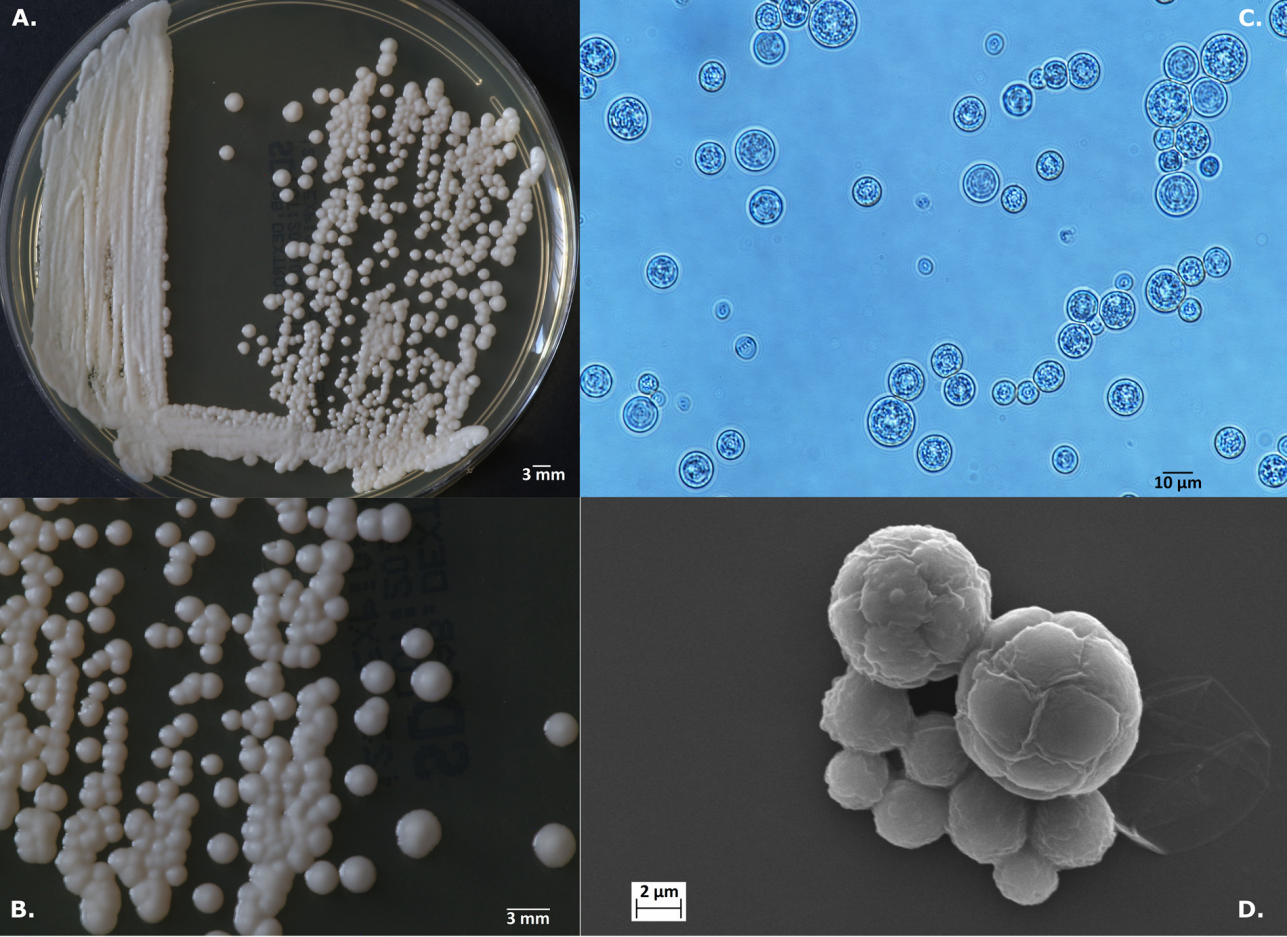
*Prototheca fontanea* sp. nov. strain PK2 type strain (T). (A and B) Colonies on SDA medium after 168 h at 25°C. (C and D) Details of cell morphology, as seen using optical microscopy (unstained, ×1,000) (C) and SEM (×6,000).

***Prototheca fontanea* Jagielski et Iskra sp. nov. (i) Description.** Sporangia and sporangiospores are globose, measuring on average 12.4 by 12.1 μm and 9.2 by 8.8 μm; capsule is absent; after a 144-h incubation on Sabouraud dextrose agar at 25°C, colonies are creamy-white, circular, raised, glistening, with smooth surface and margins, of butyrous consistency, with up to 4 mm in diameter (Fig. 8); grows at 25°C and 30°C, but poorly at 35°C; assimilates glucose, glycerol, galactose, and trehalose (Table 2).

**(ii) Holotype.** Resin-embedded EM specimen of strain PK2, deposited at the Herbarium of the Faculty of Biology at the University of Warsaw (Herbarium WA), Warsaw, Poland (type specimen no: WA0000125951).

**(iii) Type locality.** Water from the municipal fountain. Warsaw, Mazovia, Poland.

**(iv) Etymology.** The epithet *fontanea* (fon.ta’nea. L. fem. n. *fontana*, fountain, N.L. nom. fem. adj. *fontanea*, from or referring to a municipal fountain (Warsaw, Poland), from where the strain was isolated).

## DISCUSSION

The concept of the environmental ubiquity of the *Prototheca* algae has long lingered in the literature. It seems, however, to be based intuitively on the analogy with the ecological habitat of closely related *Chlorella* spp. and other green microalgae rather than on empirical evidence. This is apparent when considering the occurrence of *Prototheca* spp. in water environments. Although many *Prototheca* isolates from aquatic sources have been reported in the past (3, 17–21, 26, 27, 29–31), only a single experimental study in the late 1970s has surveyed the distribution of the algae across a wide range of aquatic ecosystems, over an extended time period, and with a well-suited methodology, recording environmental parameters and improving the algal recovery through minimizing or eliminating the growth of the contaminating microflora (9, 32).

After more than 4 decades, the present study is the first to attempt a large-scale investigation into the *Prototheca* sp. occurrence in waterbodies, both natural and artificial.

The overall prevalence of *Prototheca* spp. in waterbodies examined in this work was calculated at 14.1%. This was conspicuously lower than what was reported in the previously cited American study, where the algae were detected in every stream, river, lake, and pond sampled (22 waterbodies in total) (9, 32). The difference can be explained by at least four factors. One is an uneven proportion of the water sources of each type tested. Second, is the overrepresentation of certain types of water bodies examined (e.g., watercourses clearly predominated in the American study). Third is the geographic and climatic characteristics of the analyzed settings. In the American study, the samples were mainly collected from the eastern coast of the United States, characterized by a humid, subtropical climate. Fourth is a much more extended sampling time window in the present study. The samples were collected on 38 separate dates of 12 months, over an 18-month period, including 8 dates of 4 autumnal months and 2 dates of the winter season. In contrast, in the study of our predecessors, the environmental sites were sampled on 23 dates, only from June through September, which normally is the period of highest temperature and precipitation throughout the year in most of the temperate climate zone countries, including Poland and the United States. Both warmth and humidity are known to favor the growth of the *Prototheca* algae (1, 3, 17, 33). This is reflected in the fact that samples collected during the summer season (June to September) yielded almost twice as many *Prototheca* strains as did the samples collected during the other months of the year (30/127 or 23.6% versus 21/235 or 8.9%; *P < *0.001). This correlation might have been even stronger had we measured the temperature of our water samples. The lack of such measurements marks a limitation to our study. Subtle differences in the sample preparation procedures might also be responsible for the discrepancy of the *Prototheca* prevalence in freshwaters. Interestingly, this prevalence, determined by the current study, was not much different compared to the dairy herd environment. As assessed in several papers, the total isolation rate of *Prototheca* spp. from the immediate cattle environment ranged from 2.7% to 25% (3, 19, 20, 30, 31, 33, 34). However, in most of these studies, only less than half of the sites investigated were water related, exclusively or largely comprising watering troughs (3, 19, 31, 34).

An important observation from the study was that *Prototheca* spp. cultured from flowing waters far outnumbered those from stagnant water basins (78.4% versus 15.7%). This disparity was even more pronounced upon comparison of the fractions of the *Prototheca*-positive samples among all samples of the two groups of waterbodies (35.1% versus 4.9%; *P < *0.001). Furthermore, the proportion of the *Prototheca* strains isolated from sediment samples was over twice as high for flowing than for standing waters (35.7% versus 15.4%; *P > *0.1). These findings fully corroborate those from 40 years ago (9, 32). The only exception was that, according to the old report, the sediment of still-water ecosystems contained twice as many *Prototheca* spp. as that from running waterbodies (9). This was not observed in our study, and a possible explanation for this is that the sediment in the earlier study might have been gathered chiefly from the floor of the waterbody (bottom sediment), while here, we mostly collected floodplain and overbank sediments.

Still, the general picture which emerges from our study and depicts the *Prototheca* lifestyle in aquatic habitats fits well into the scenario proposed earlier in which *Prototheca* spp. are bottom dwellers, where they attach to sand, vegetation, and other substrates, and when only sediment is disturbed by turbulence due to natural events (wind, rain, swell, currents) or animal and human activity (e.g., inflows of domestic and industrial effluents, vessel traffic, riparian cattle grazing), the algae can be resuspended into the overlying water column and transported downstream. Furthermore, it has been hypothesized that *Prototheca* spp. are not autochthonous to aquatic habitats but are transients arising from external sources with rainfall washes. This view was supported, first, by a positive correlation between precipitation and *Prototheca* occurrence and, second, by the fact that the algae could not maintain their growth when experimentally cultured in stream water chambers (9). In this study, slightly less than half (45.1%) of the *Prototheca* isolates were retrieved from areas that had experienced rainfall at least once in the 72 h prior to sampling (data not shown). In all of the *Prototheca-*positive sampling sites, the rainfall was rather light, with a precipitation rate of <14 mm in 1 day. This inclines us to the conclusion, which had already been reached (9), that the *Prototheca* spp. enter the aquatic systems through point pollution sources, primarily domestic and municipal wastewater discharge.

In our study, the *Prototheca* isolation rate among samples collected in urban areas was 4-fold higher than that among samples of rural origin (15.9% versus 3.6%; *P = *0.01). Municipalities, and especially, highly industrialized and densely populated metropolitan areas are the leading producers of wastewater being disposed, as either point- or dispersed-source pollutants, from households and transport, public utility, and industrial infrastructure. It is thus not surprising that nearly half (45.1%) of the *Prototheca* isolates were recovered from the Vistula River, along 57 km of its corridor across Warsaw and Cracow, the two largest population agglomerations in Poland. The Vistula River, the longest river in the country, serves as a major source of water for domestic and industrial use, but it is also a major receiver of domestic and industrial effluents.

For the Vistula River, the overall organic pollution, expressed by the total organic carbon (TOC) and biochemical oxygen demand (BOD) concentrations, is relatively low, with the two indicators not exceeding, on average, 10 mg C/L and 6 mg O_2_/L, respectively (35, 36). In this study, water samples from the Vistula River were taken in those sections (lower and middle) of its course for which both TOC and BOD levels approximate the highest values reported as mentioned above. It is possible that had we included samples from other sections of the river, less burdened with organic load, we might have achieved a much lower yield of *Prototheca* spp. Organic pollution has been considered a pivotal factor influencing the occurrence of *Prototheca* spp. in the environment. In general, the high organic compound load (TOC, BOD) enhanced the *Prototheca* isolation (9, 29, 32, 37). This correlation was also observed for stagnant waters and exemplified, to some extent, in the present study. Of the 49 natural freshwater reservoirs (lakes, ponds) sampled, the only two (Lake Mikołajskie and Służewiecki Pond), where *Prototheca* spp. were isolated have been severely exposed to heavy organic loading, albeit from different sources. Lake Mikołajskie has been regularly contaminated by a long-term inflow of pollutants from excessive tourism and agriculture (38). Moreover, Lake Mikołajskie is connected with Tałty Lake, into which untreated wastewater flows from an occasionally malfunctioning local automatic-biological sewage treatment plant (39). However, Służewiecki Pond is a eutotrophic, seminatural park pond whose pollution is mainly due to inflow from the Służewiecki Stream, the largest tributary of the River Vistula within the Warsaw agglomeration area. The stream, which itself was a source of *Prototheca* algae, starts its flow in the industrial district of Włochy, in which Warsaw Chopin Airport is located, and then runs through an intensively built-up residential area, thus carrying a considerable amount of industrial and domestic discharge (40, 41).

The least *Prototheca*-abundant were artificial waterbodies, comprising 18 reservoirs varying in size, morphology, hydroperiod, and functionality. Typically, artificial waterbodies are small, shallow, and lentic or semilentic impermanent environments, subject to a strong anthropogenic pressure. They are often embedded in heavily exploited landscapes, exposing them to increased runoff of agrochemicals (fertilizers and pesticides) and urban waste. The changeability of abiotic conditions and high vulnerability to anthropogenic stressors make artificial waters unstable ecosystems, adversely affecting their biodiversity (42). The paucity of protothecae in these environments might be linked to an excessive accumulation of toxic organic pollutants and/or diurnal dynamics of water temperature (e.g., fast warming during intense sunshine). The latter may not be the case, at least for some *Prototheca* species, since the algae could be isolated from the thermally polluted Żerański Canal, which is a part of the Warsaw Żerań Power Station cooling system and links the Vistula River with the Zegrze Reservoir on the River Narew.

However, without specific analytical tests aimed at establishing the gross amount of organic matter in water samples, the conclusions about the prevalence of *Prototheca* spp. in different waterbodies remain largely conjectural and speculative.

At the species level, the three most frequently isolated species in this study were *P. wickerhamii* (33%), *P. pringsheimii* (23%), and *P. cerasi* (14%). *P. wickerhamii* is a well-established species whose occurrence in aquatic environments has been reported as ubiquitous. The alga is highly prevalent in domestic and municipal sewage, from which it flows into natural water bodies and may subsequently contaminate potable water and food (9). Notably, *P. wickerhamii* is a predominant etiological agent of human protothecosis, and exposure to contaminated water is a major route of infection with this pathogen (1, 2).

Far less explored is the ecology of *P. pringsheimii* and *P. cerasi*, two newly designated species with single or very few members, mostly of unspecified provenance (5). The type strain of *P. pringsheimii* is thought to derive from a lichen (Xanthoria parietina), while so far, the only representative of *P. cerasi* originates from the bark of a cherry tree in Japan (5). This study is thus the first to show the aquatic reservoir for those two species.

Only a minor part (15.7% in total) of the cultured *Prototheca* algae was represented by *P. bovis* and *P. ciferrii*, both of which have been associated with the dairy herd environment, with the former species being implicated in the vast majority of cases of bovine mammary protothecosis. Studies investigating the cow barn surroundings have repeatedly detected *P. bovis* and *P. ciferrii* in drinking water samples, with contrasting proportions of the two species (3, 20, 30, 31). Also, the frequency of isolation of both species from water samples differed between the studies, with 5.2% in Japan and four times that (21.4%) in Poland (3, 30). At the same time, the overall isolation rate of both *P. bovis* and *P. ciferrii* from the immediate cattle environment ranged from 2.7% to 19.3% (3, 20, 29, 30, 33). The corresponding rate calculated for this study (8/362 or 2.2%) lends further support for a close association of the two species with dairy farming and cow barn sources.

The three newly described species were isolated either from the urban Vistula River corridor (*P. vistulensis*, *P. lentecrescens*) or a municipal fountain (*P. fontanea*), suggesting that they plausibly originated from anthropogenic inputs. Additionally, *P. vistulensis* and *P. lentecrescens* were collected at the river banks near heavily trafficked road bridges, where drainage outlets deliver polluted runoff directly into the river water. This, together with the fact that all but two (49/51; 96%) *Prototheca* strains cultured derived from urbanized areas, implies that the environmental prevalence of the protothecae is firmly impacted by anthropogenic activities and that the metropolitan landscape provides conditions conducive for the growth of the algae.

The bulk of the *Prototheca* strains (78.4%) were isolated from flowing waters, including all strains of *P. ciferrii*, *P. cookei*, *P. lentecrescens*, *P. vistulensis*, and *P. zopfii* and all but one strain of *P. cerasi*. Many more isolates are needed to determine whether these species are particularly restricted to flowing waters. Still, the results of this study conspicuously show that the *Prototheca* algae are much less adapted to stagnant waters, which adheres to previous observations (9, 32).

Given a relatively low recovery rate, the species diversity of the *Protothecae* was remarkable. The previous complexity of the genus might have been underestimated largely due to insufficient, poorly discriminatory diagnostic tools.

The description of new *Prototheca* species has prompted us to further develop the previously designed PCR-RFLP scheme for identifying species of *Prototheca* algae, based on the *CYTB* gene marker (5, 43). In the revised scheme, *P. lentecrescens* and *P. fontanea* produced unique restriction patterns upon RsaI/TaiI digestion, whereas *P. vistulensis* separated from *P. zopfii* only after secondary restriction with VspI. Moreover, we have included in our refined diagnostic algorithm another recently described species, *P. paracutis*, providing its differentiation from *P. cutis* through BfaI digestion (Fig. 5). The complete algorithm for *Prototheca* sp. identification is depicted in Fig. 6.

To conclude, after 4 decades, this study is the first to explore the occurrence of *Prototheca* spp. in water ecosystems. The study points to four major findings. First, the *Prototheca* isolation rate was relatively low, which contradicts the widely held view of their ubiquity, which is perpetuated in the literature. Second, *Prototheca* spp. were most prevalent in rivers of urbanized areas, indicating that the algae are primarily adapted to lotic ecosystems with a high input of organic matter. Importantly, the occurrence of *Prototheca* spp. seems to be influenced by not only the content of organic matter, but also its quality and potential toxicity. Third, finding three novel species among rather a scarce sampling of strains recovered speaks of important species diversity of the *Prototheca* algae. Finally, a new, revised scheme for the identification of all *Prototheca* species so far described was provided.

## MATERIALS AND METHODS

### Sampling, isolation, and culture conditions.

**FIG 9:**
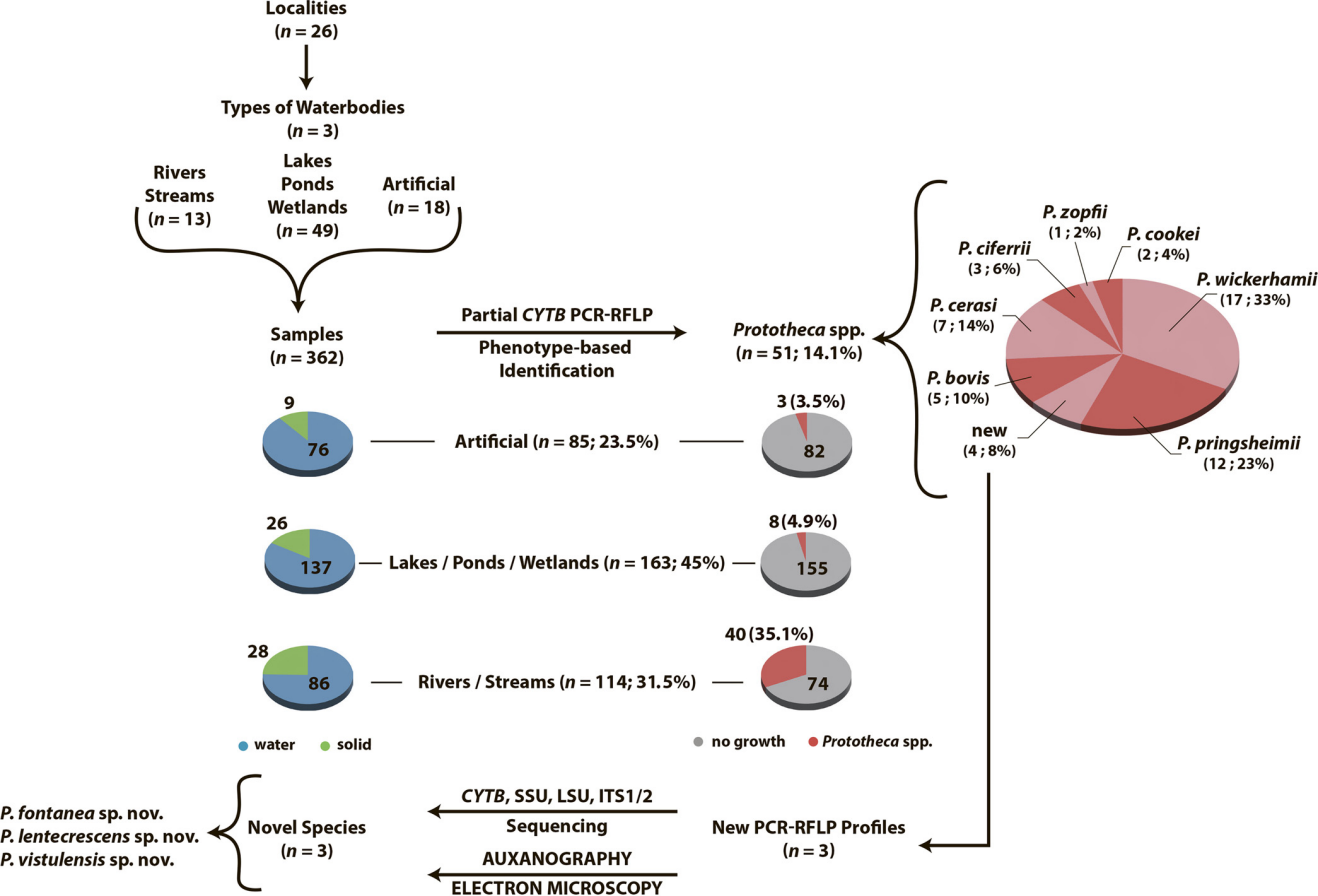
Flowchart depicting the sampling strategy and identification procedure of cultured *Prototheca* isolates. Auxanography was performed with API ID32C identification strips for yeasts (bioMérieux, Marcy-l'Etoile, France).

**FIG 10:**
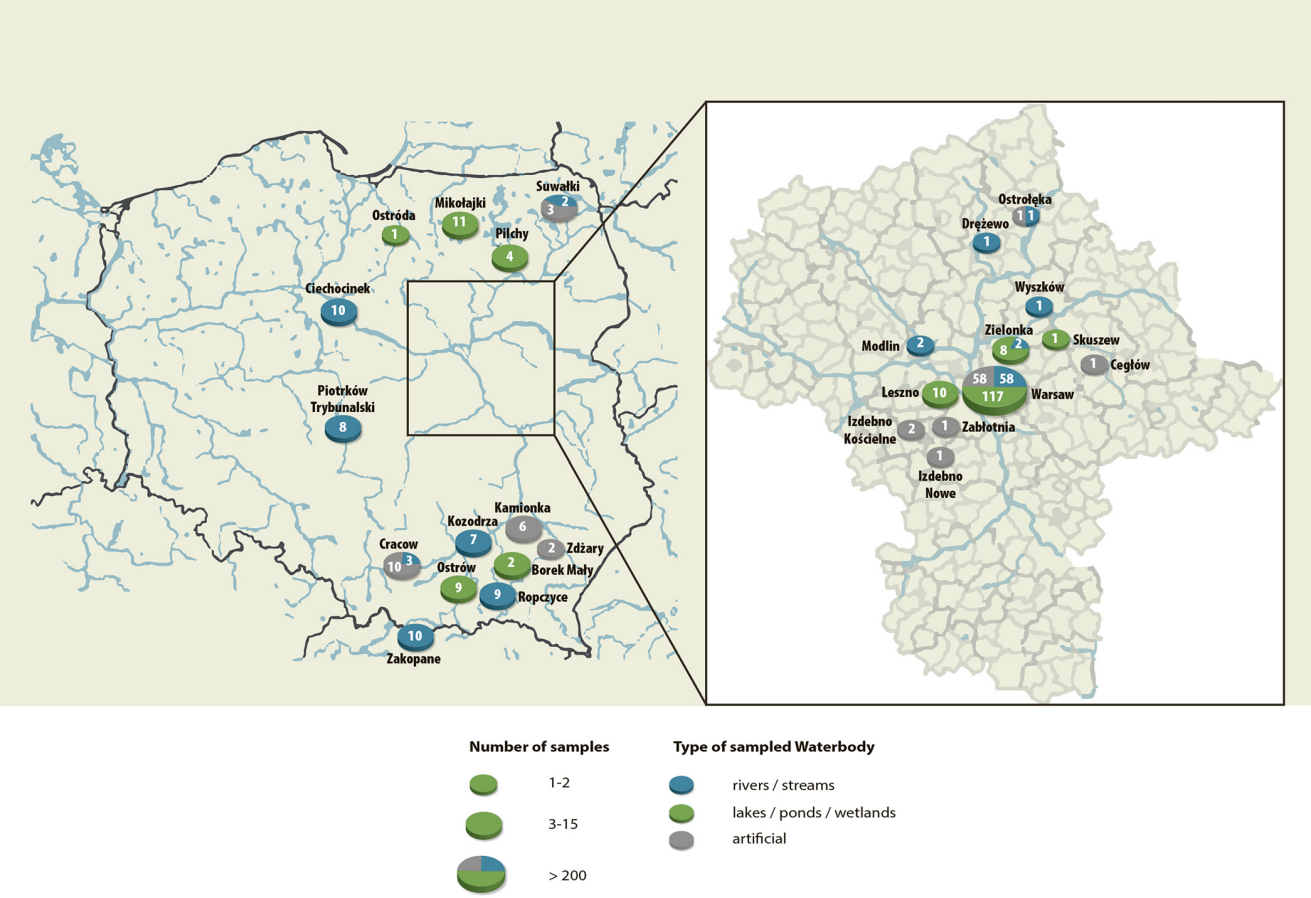
Map of Poland with major waterbodies (in blue) and localities investigated. The enlarged is a map of Mazovia, the largest province of Poland.

The study was performed over an 18-month period (i.e., from 1 October 2018 to 31 March 2020). A total of 362 samples were collected from 62 natural (277 samples) and 18 artificial (85) water reservoirs at 88 sampling sites in 26 localities across 7 out of 16 voivodships of Poland, mostly in the central part of the country. The strategy of sampling and identification are shown in Fig. 9. The geographic positions of the localities investigated in this study and the categories of the collected specimens are shown in Fig. 10 and Table 1.

Both standing (lakes, ponds, marshes) and flowing (rivers, streams) freshwater sources were sampled. Among the artificial water reservoirs were canals, ditches, moats, retention basins, municipal ponds, and fountains (Table S1). Both aqueous and solid samples were taken, with the latter including soil, mud, sand, and coastal and bottom sediments, as well as decaying vegetation in the immediate area of the water source.

Liquid samples were collected using sterile 50-mL plastic tubes lowered into the water upside down obliquely, turned upward at a depth of ca. 10 to 30 cm, and pulled out to retrieve the sample. For shallow-water reservoirs, the tubes were inserted horizontally into the water to avoid disturbing the bottom. Solid and semisolid specimens were collected in sterile plastic containers with the V-shaped spatulas attached to the containers’ inner lids.

The samples, at least two per each site, were transported to the laboratory for microbiological analysis. Liquid and semisolid samples (aliquots of 0.1 mL) were spread on *Prototheca* isolation medium (PIM) (43) agar plates, either after concentration through centrifugation (12,000 relative centrifugal force [rcf], 10 min) and resuspension of the sediment in 1 mL of sterile water or after preincubation in liquid PIM. Enrichment of samples with 5 mL of liquid PIM for 48 h at 30°C enhanced the detection and recovery of algae, preventing them from being overgrown by contaminating bacteria and/or fungi. Solid specimens were ground in a sterile porcelain mortar in sterile water, preincubated in liquid PIM for 48 h at 30°C, and then plated and incubated as described above.

Every sample collected and its laboratory variant (i.e., concentrated or preincubated in liquid PIM) was cultured in duplicate. Plates were incubated at 30°C for 2 to 5 days. Pure cultures were maintained on Sabouraud dextrose agar (SDA, Becton, Dickinson, Franklin Lakes, NJ, USA) plates at 30°C, aerobically in the dark.

Colonies suspected to be *Prototheca* spp. were subcultured on SDA and subjected to generic identification, on the basis of macro- and micromorphology, as evaluated using a Primo Star light microscope (Zeiss, Oberkochen, Germany).

### Molecular typing and sequencing.

DNA isolation. Species-level identification was achieved by molecular typing with the partial *CYTB* gene as a marker (44). Preparation of genomic DNA was performed as described elsewhere (45). Briefly, a loopful of cells from SDA plates was suspended in the β-mercaptoethanol-lyticase-proteinase K-containing buffer in a tube with 1- to 2-mm glass beads. The suspension was vortexed vigorously and centrifuged, and the aqueous phase was transferred to a fresh tube, prior to DNA extraction using the GeneMATRIX environmental DNA and RNA purification kit (EURx, Gdańsk, Poland), as per the manufacturer’s instructions. The purified DNA, dissolved in TE buffer (10 mM Tris-HCl, 1 mM EDTA, pH 8.0), was quantified with the NanoDrop ND-1000 spectrophotometer (Thermo Fisher Scientific, Waltham, MA, USA) and used as a template for PCR amplification or stored at −20°C until use.

**PCR amplification and PCR-restriction fragment length polymorphism (PCR-RFLP) analysis.** For amplification of the partial *CYTB* gene, the primer pair *cytb*-F1 and *cytb*-R2 was used (Table S2) (44). PCR mixtures (20 μL) contained 0.2 μM each primer, approximately 10 ng of template DNA, and 0.5 U of OptiTaq DNA polymerase (EURx, Gdańsk, Poland). Thermocycling conditions were 3 min at 95°C, followed by 35 cycles of 30 s at 95°C, 30 s at 50°C, and 30 s at 72°C, with a final extension of 5 min at 72°C.

PCR products (5 μL) were visualized on ethidium bromide (EtBr)-stained 0.7% (wt/vol) agarose-TBE gels and double-digested with FastDigest enzymes RsaI and TaiI (Thermo Fisher Scientific, Waltham, MA, USA). Restriction reaction mixtures contained 1.5 μL of FastDigest buffer, 10 μL of PCR product, and 0.5 μL of each enzyme in a final volume of 15 μL. Digestions were performed at 37°C for 5 min. The restriction products were separated on 4% (wt/vol) agarose-TBE gels and visualized by EtBr staining. Analysis of the electropherograms was done with the UVP BioDoc-IT imaging system (Analityk Jena, Jena, Germany). Additional restriction with MfeI was performed to further distinguish between *P. cookei* and *P. pringsheimii*. The MfeI digestion was carried out under the same conditions as for RsaI/TaiI double digestion.

Species-level assignment was based on analysis of banding patterns according to an algorithm described elsewhere (5).

**Sequencing.** PCR-sequencing of the *CYTB* gene was performed for 4 strains, whose RsaI/TaiI RFLP patterns either failed to match any known patterns (PK1, PK2, PK6) as per the identification algorithm or did match a pattern of the previously described species, yet the strain’s morphology was clearly different (W3). The *CYTB* amplicons were purified using the short DNA clean-up purification kit (A&A Biotechnology, Gdynia, Poland) and subjected directly to Sanger sequencing with the same primers as those used for the amplification. The obtained sequences were subjected to a BLAST search against the Prototheca-ID curated database (46) and GenBank database, with default settings.

For three *Prototheca* strains (PK1, PK2, W3) with sequence similarity of their *CYTB* genes below 95.4%, three rDNA loci, the entire 18S small-subunit (SSU) rRNA gene, the two variable domains (D1/D2) at the 5′ end of the 28S large-subunit (LSU) rRNA gene, and the two internal transcribed spacer (ITS1/2) regions, were PCR-amplified with the primers and conditions described previously (44). All PCR products were confirmed by gel electrophoresis on EtBr-stained 0.7% (wt/vol) agarose-TBE gels.

The SSU, LSU, and ITS1/2 amplicons were cloned into the pPCR-Script Amp SK (+) cloning vectors using the PCR-Script Amp cloning kit (Stratagene, La Jolla, CA, USA) according to the manufacturer’s instructions, and the Qiagen plasmid minikit (Qiagen, Valencia, CA, USA) was used for isolation of plasmids from single, insert-positive clones. The inserts were then sequenced with the vector- and insert-specific primers listed in Table S2 on an Applied Biosystems 3730xl genetic analyzer, using the BigDye v3.1 chemistry and dGTP BigDye Terminator v3.0 chemistry mixed in a 1:1 ratio (Applied Biosystems, Foster City, CA, USA).

FinchTV v1.4.0 software (Geospiza, Akron, OH, USA) and SeqMan Pro v9.1 software (DNAStar, Madison, WI, USA) were used for analysis of sequencing data and consensus sequence assembly, respectively. The *CYTB* gene and rDNA sequences determined in this study were deposited in the GenBank database under the accession numbers provided in Table 2.

All *Prototheca* strains, under appropriate species assignations, were preserved in Viabank vials (Medical Wire & Equipment, Corsham, UK) and stored at −70°C. The strains are part of the culture collection of the Department of Medical Microbiology, Faculty of Biology, University of Warsaw, and are available upon request.

In addition, holotypes of the new species were prepared as resin-embedded EM specimens and deposited in the Herbarium of the Faculty of Biology, University of Warsaw (Herbarium WA), Warsaw, Poland, as required by the International Code of Nomenclature for algae, fungi, and plants (47).

**Phylogenetic analysis.** Sequences of the *CYTB* gene and rDNA genes (SSU, LSU, ITS1/2) of *Prototheca* strains from this study were aligned with the corresponding sequences of other *Prototheca* spp. and other Chlorophyta algae (*Auxenochlorella*, *Chlamydomonas*, *Chlorella*, and *Helicosporidium*), retrieved from the GenBank database, using MAFFT software v7.310 with default settings (48). Regions with ambiguous homology were excluded using trimAl v1.3 with a gap threshold set at 0.3 and a similarity threshold set at 0.001 (49). Phylogenetic trees were constructed using the IQ-TREE webserver (http://iqtree.cibiv.univie.ac.at/) under automatic substitution model with ultrafast bootstrap analysis with a total of 1,000 random replicates (50). Pairwise-identity matrices were generated in MEGA v7.0.26, based on alignments with the regions of uncertain homology removed, using a simple number of differences model (51).

### Phenotypic characterization of new species.

Strains of *Prototheca* spp. classified into the newly proposed species were subjected to a detailed phenotypic analysis, including macro- and micromorphology examination, assessment of growth rates at different temperatures, and auxanographic carbohydrate assimilation tests. The morphological features of *Prototheca* cells were investigated using a NIKON Eclipse E-600 light microscope (Nikon Instruments Co., Tokyo, Japan) on direct, wet-mount (0.85% NaCl) smears from culture, unstained or negatively stained with 10% nigrosine. Photographs were acquired using a Nikon DX-1200 digital camera connected to the microscope. NIS Elements BR software (https://www.r-project.org/) was used for morphometric studies on the images captured beforehand. To determine cell sizes, cell lengths (i.e., the longest dimensions) and cell widths (i.e., the shortest dimension) were measured, and the average dimensions from 10 independent measurements were assessed. *Prototheca* cells were selected randomly at various phases of development. Cells within both the sporangia and the released endospores were taken for the measurements.

Scanning electron microscopy (SEM) images were acquired and analyzed using Axiovision v4.8 software (Carl Zeiss, Thornwood, NY, USA) and LEO-32 software (Carl Zeiss, Oberkochen, Germany), respectively.

Single-colony measurements were made from digital images using ImageJ software (https://imagej.nih.gov/ij/).

For temperature tests, each strain was subcultured onto SDA medium and incubated at 25°C, 30°C, and 35°C for up to 7 days, with readings taken every 24 h. Each strain was tested in triplicate.

The assimilation profiles were examined using the API ID32C system (bioMérieux, Marcy-l’Étoile, France) according to the manufacturer’s instructions. Readings were taken daily from 24 to 72 h, with each test repeated three times.
