# Supplementary material for: Occurrence of Prototheca Microalgae in Aquatic Ecosystems with a Description of Three New Species, Prototheca fontanea, Prototheca lentecrescens, and Prototheca vistulensis
Source: Appl Environ Microbiol. 2022 Oct 27;88(22):e01092-22. doi: 10.1128/aem.01092-22 (PMC9680628; doi:10.1128/aem.01092-22)
Supplement: Supplemental file 1 — Fig. S1 to S3 and Tables S2 and S3. Download aem.01092-22-s0001.pdf, PDF file, 0.5 MB [file aem.01092-22-s0001.pdf]

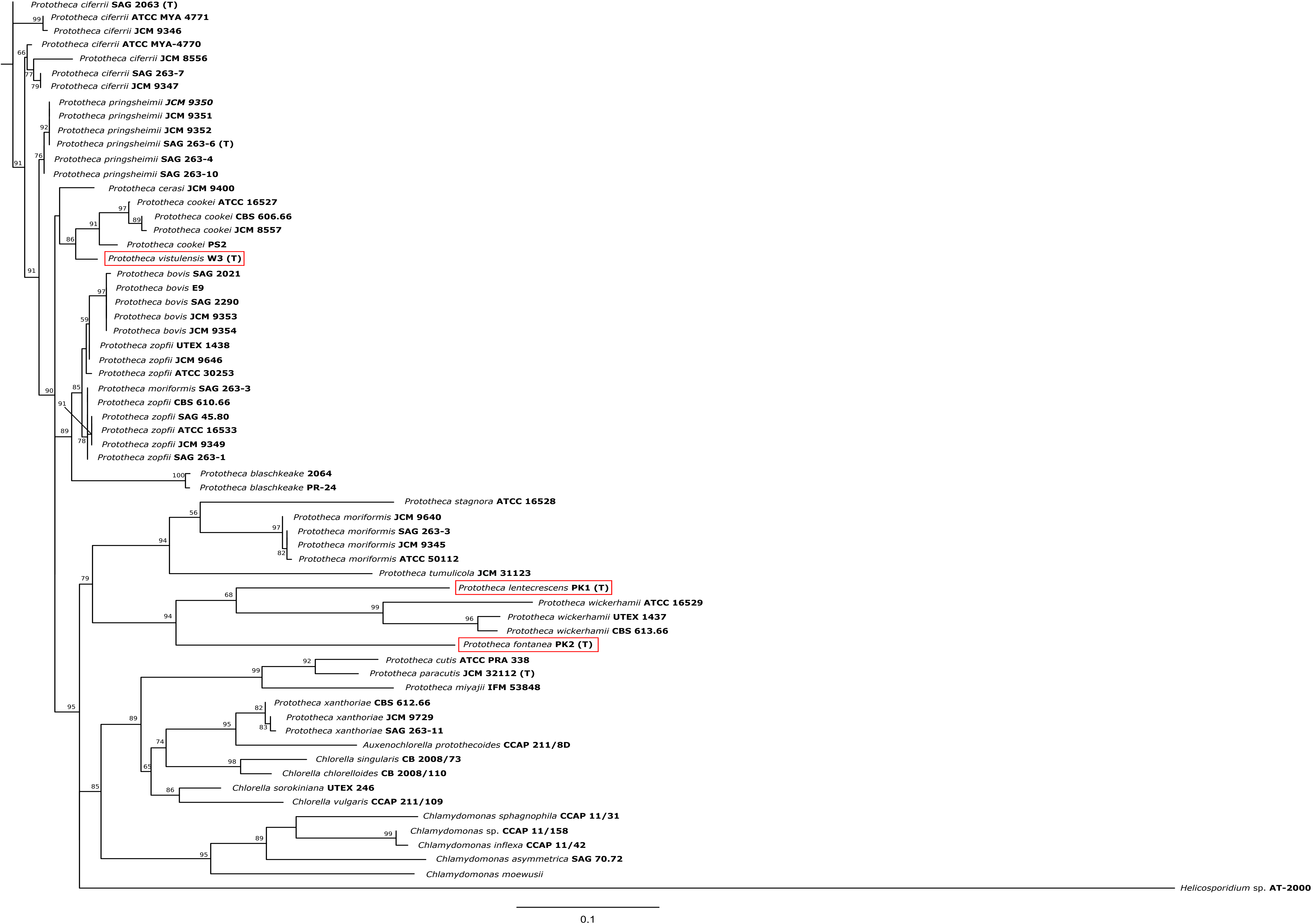
Prototheca ciferrii SAG 2063 (T)
Prototheca ciferrii ATCC MYA 4771
Prototheca ciferrii JCM 9346
Prototheca ciferrii ATCC MYA-4770
Prototheca ciferrii JCM 8556
Prototheca ciferrii SAG 263-7
Prototheca ciferrii JCM 9347
Prototheca pringsheimii JCM 9350
Prototheca pringsheimii JCM 9351
Prototheca pringsheimii JCM 9352
Prototheca pringsheimii SAG 263-6 (T)
Prototheca pringsheimii SAG 263-4
Prototheca pringsheimii SAG 263-10
Prototheca cerasi JCM 9400
Prototheca cookei ATCC 16527
Prototheca cookei CBS 606.66
Prototheca cookei JCM 8557
Prototheca cookei PS2
Prototheca vistulensis W3 (T)
Prototheca bovis SAG 2021
Prototheca bovis E9
Prototheca bovis SAG 2290
Prototheca bovis JCM 9353
Prototheca bovis JCM 9354
Prototheca zopfii UTEX 1438
Prototheca zopfii JCM 9646
Prototheca zopfii ATCC 30253
Prototheca moriformis SAG 263-3
Prototheca zopfii CBS 610.66
Prototheca zopfii SAG 45.80
Prototheca zopfii ATCC 16533
Prototheca zopfii JCM 9349
Prototheca zopfii SAG 263-1
Prototheca blaschkeake 2064
Prototheca blaschkeake PR-24
Prototheca stagnora ATCC 16528
Prototheca moriformis JCM 9640
Prototheca moriformis SAG 263-3
Prototheca moriformis JCM 9345
Prototheca moriformis ATCC 50112
Prototheca tumulicola JCM 31123
Prototheca lentecrescens PK1 (T)
Prototheca wickerhamii ATCC 16529
Prototheca wickerhamii UTEX 1437
Prototheca wickerhamii CBS 613.66
Prototheca fontanea PK2 (T)
Prototheca cutis ATCC PRA 338
Prototheca paracutis JCM 32112 (T)
Prototheca miyajii IFM 53848
Prototheca xanthoriae CBS 612.66
Prototheca xanthoriae JCM 9729
Prototheca xanthoriae SAG 263-11
Auxenochlorella protothecoides CCAP 211/8D
Chlorella singularis CB 2008/73
Chlorella chlorelloides CB 2008/110
Chlorella sorokiniana UTEX 246
Chlorella vulgaris CCAP 211/109
Chlamydomonas sphagnophila CCAP 11/31
Chlamydomonas sp. CCAP 11/158
Chlamydomonas inflexa CCAP 11/42
Chlamydomonas asymmetrica SAG 70.72
Chlamydomonas moewusii
Helicosporidium sp. AT-2000
0.1

Suppl. Figure 1. Phylogenetic tree constructed through maximum likelihood analysis based on ITS sequences. The bootstrap values obtained by the analysis are marked at the nodes.

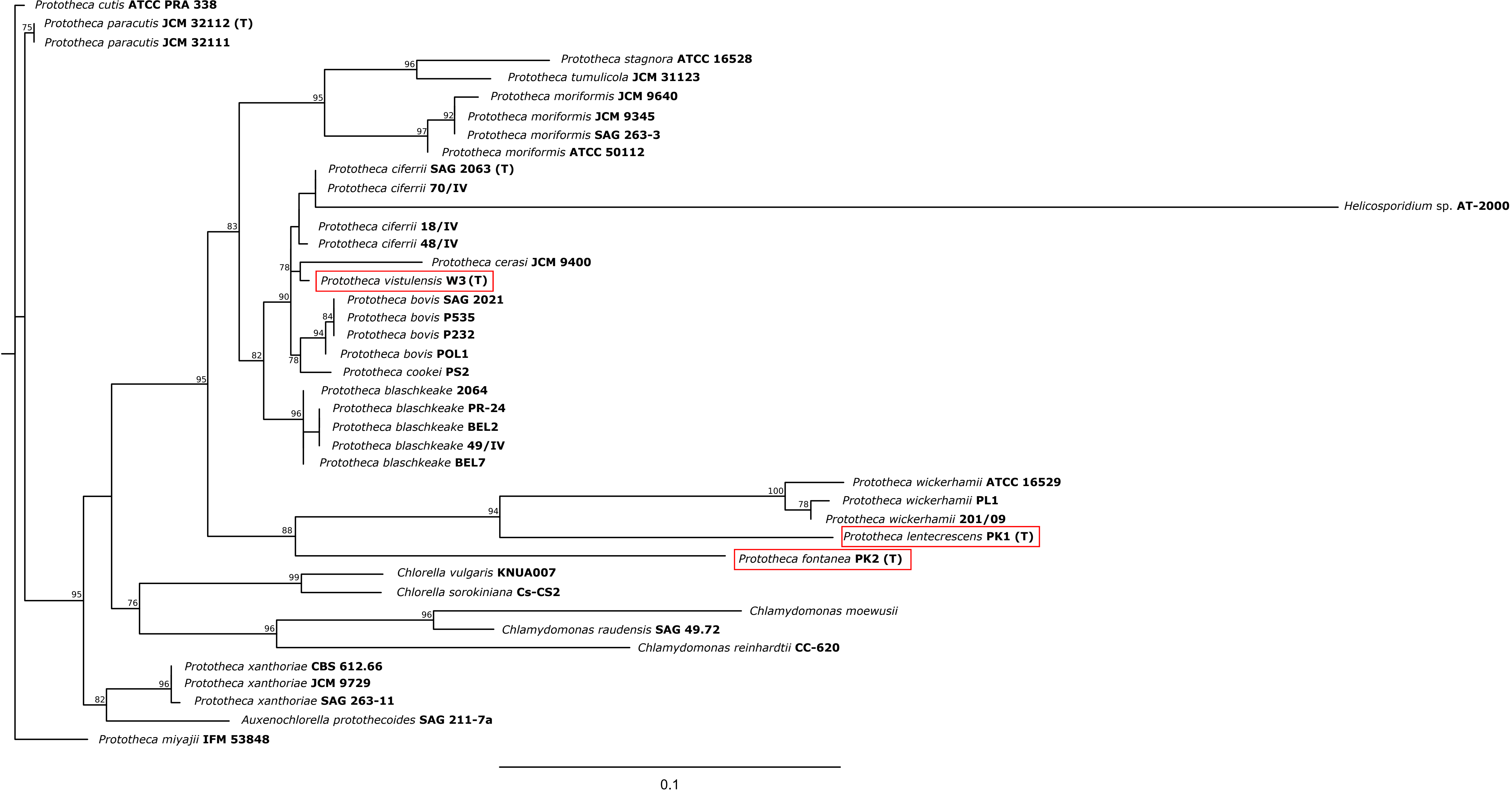

Prototheca cutis ATCC PRA 338
Prototheca paracutis JCM 32112 (T)
Prototheca paracutis JCM 32111
Prototheca stagnora ATCC 16528
Prototheca tumulicola JCM 31123
Prototheca moriformis JCM 9640
Prototheca moriformis JCM 9345
Prototheca moriformis SAG 263-3
Prototheca moriformis ATCC 50112
Prototheca ciferrii SAG 2063 (T)
Prototheca ciferrii 70/IV
Helicosporidium sp. AT-2000
Prototheca ciferrii 18/IV
Prototheca ciferrii 48/IV
Prototheca cerasi JCM 9400
Prototheca vistulensis W3 (T)
Prototheca bovis SAG 2021
Prototheca bovis P535
Prototheca bovis P232
Prototheca bovis POL1
Prototheca cookei PS2
Prototheca blaschkeake 2064
Prototheca blaschkeake PR-24
Prototheca blaschkeake BEL2
Prototheca blaschkeake 49/IV
Prototheca blaschkeake BEL7
Prototheca wickerhamii ATCC 16529
Prototheca wickerhamii PL1
Prototheca wickerhamii 201/09
Prototheca lentecrescens PK1 (T)
Prototheca fontanea PK2 (T)
Chlorella vulgaris KNUA007
Chlorella sorokiniana Cs-CS2
Chlamydomonas moewusii
Chlamydomonas raudensis SAG 49.72
Chlamydomonas reinhardtii CC-620
Prototheca xanthoriae CBS 612.66
Prototheca xanthoriae JCM 9729
Prototheca xanthoriae SAG 263-11
Auxenochlorella protothecoides SAG 211-7a
Prototheca miyajii IFM 53848
75
96
95
92
97
83
78
90
84
94
82
78
96
95
100
78
94
88
99
76
96
96
95
96
82
0.1

Suppl. Figure 2. Phylogenetic tree constructed through maximum likelihood analysis based on D1/D2 LSU sequences. The bootstrap values obtained by the analysis are marked at the nodes.

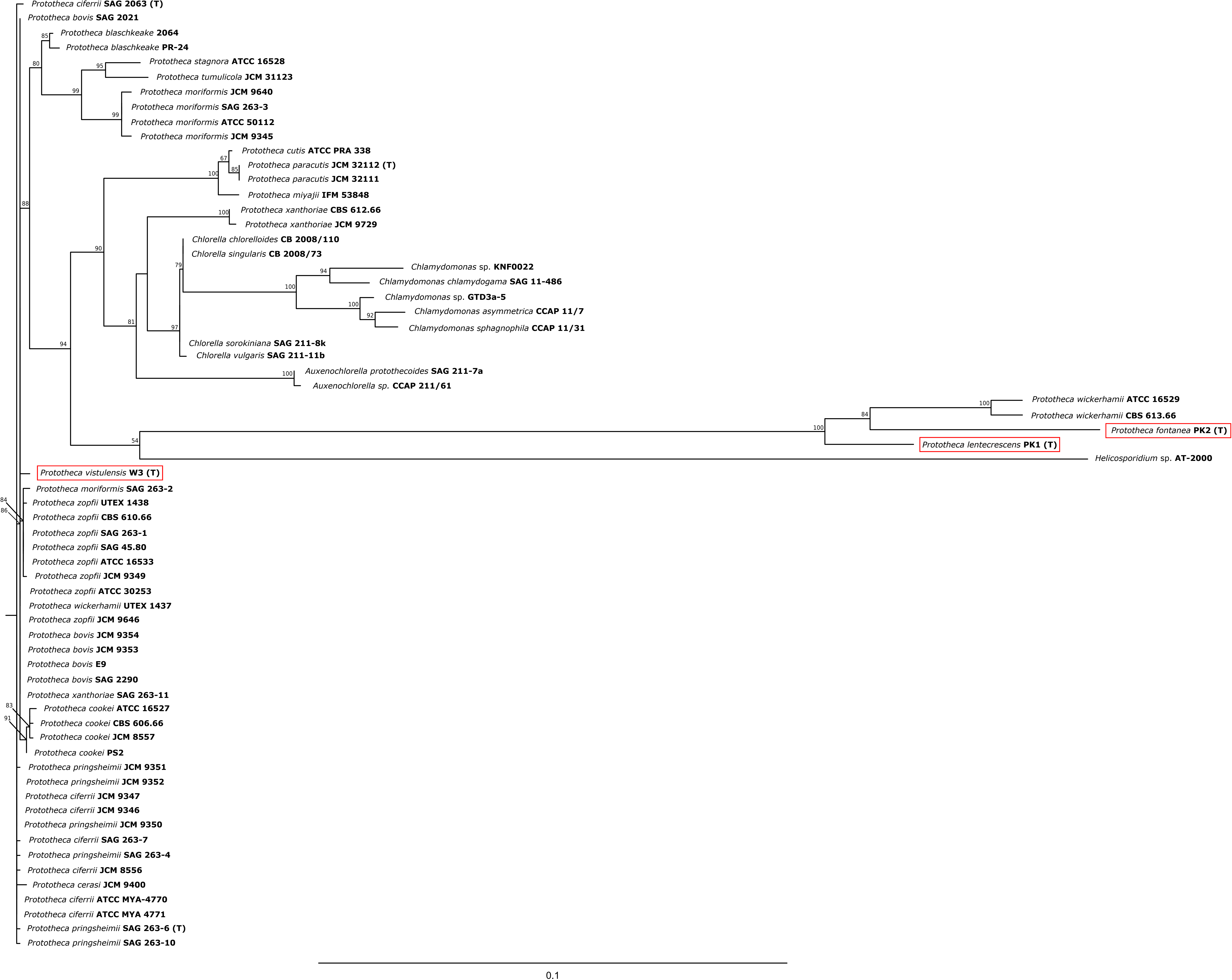

Prototheca ciferrii SAG 2063 (T)
Prototheca bovis SAG 2021
Prototheca blaschkeake 2064
Prototheca blaschkeake PR-24
Prototheca stagnora ATCC 16528
Prototheca tumulicola JCM 31123
Prototheca moriformis JCM 9640
Prototheca moriformis SAG 263-3
Prototheca moriformis ATCC 50112
Prototheca moriformis JCM 9345
Prototheca cutis ATCC PRA 338
Prototheca paracutis JCM 32112 (T)
Prototheca paracutis JCM 32111
Prototheca miyajii IFM 53848
Prototheca xanthoriae CBS 612.66
Prototheca xanthoriae JCM 9729
Chlorella chlorelloides CB 2008/110
Chlorella singularis CB 2008/73
Chlamydomonas sp. KNF0022
Chlamydomonas chlamydogama SAG 11-486
Chlamydomonas sp. GTD3a-5
Chlamydomonas asymmetrica CCAP 11/7
Chlamydomonas sphagnophila CCAP 11/31
Chlorella sorokiniana SAG 211-8k
Chlorella vulgaris SAG 211-11b
Auxenochlorella protothecoides SAG 211-7a
Auxenochlorella sp. CCAP 211/61
Prototheca wickerhamii ATCC 16529
Prototheca wickerhamii CBS 613.66
Prototheca fontanea PK2 (T)
Prototheca lentecrescens PK1 (T)
Helicosporidium sp. AT-2000
Prototheca vistulensis W3 (T)
Prototheca moriformis SAG 263-2
Prototheca zopfii UTEX 1438
Prototheca zopfii CBS 610.66
Prototheca zopfii SAG 263-1
Prototheca zopfii SAG 45.80
Prototheca zopfii ATCC 16533
Prototheca zopfii JCM 9349
Prototheca zopfii ATCC 30253
Prototheca wickerhamii UTEX 1437
Prototheca zopfii JCM 9646
Prototheca bovis JCM 9354
Prototheca bovis JCM 9353
Prototheca bovis E9
Prototheca bovis SAG 2290
Prototheca xanthoriae SAG 263-11
Prototheca cookei ATCC 16527
Prototheca cookei CBS 606.66
Prototheca cookei JCM 8557
Prototheca cookei PS2
Prototheca pringsheimii JCM 9351
Prototheca pringsheimii JCM 9352
Prototheca ciferrii JCM 9347
Prototheca ciferrii JCM 9346
Prototheca pringsheimii JCM 9350
Prototheca ciferrii SAG 263-7
Prototheca pringsheimii SAG 263-4
Prototheca ciferrii JCM 8556
Prototheca cerasi JCM 9400
Prototheca ciferrii ATCC MYA-4770
Prototheca ciferrii ATCC MYA 4771
Prototheca pringsheimii SAG 263-6 (T)
Prototheca pringsheimii SAG 263-10
0.1

Suppl. Figure 3. Phylogenetic tree constructed through maximum likelihood analysis based on SSU rDNA sequences. The bootstrap values obtained by the analysis are marked at the nodes.

Supplementary Table 2. Primers used for PCR amplification and sequencing of the partial *CYTB* gene and rDNA loci.

Locus	Primer[a] designation	Primer[a] nucleotide sequence (5'→3')	Product size [bp]	Reference
CYTB	*cytb*-F1	GyGTwGAACAyATTATGAGAG	*ca*. 650	(Jagielski *et al.*, 2019a)
	cytb-R2	wACCCATAArAArTACCATTCWGG		
SSU	SSU-F1*	AACCTGGTTGATCCTGCCAGTAGTC	1808–2164	(Ye *et al.*, 2012)
	SSU-R1*	TGATCCTTCTGCAGGTTCACCTACG		
	21M13F**	TGTAAAACGACGGCCAGT		-
	M13Rev**	CAGGAAACAGCTATGACC		
	SSU3**	GCCTGAGAAACGGCTACCAC		(Masuda *et al.*, 2020)
	SSU5**	GTGGTAGCCGTTTCTCAGGC		
	SSU_F2**	TGTCAGAGGTGAAATTCTTGG		(Jagielski *et al.*, 2019a)
LSU	28S-F1	AAGCATATCAATAAGCGGAGG	*ca*. 750	(Jagielski *et al.*, 2019a)
	653	GGTCCGTGTTTCAAGACGG		
ITS	ITS4*	TCCTCCGCTTATTGATATGC	744–4437	(Lemaire *et al.*, 2011)
	ITS5*	GGAAGTAAAAGTCGTAACAAGG		
	21M13F**	TGTAAAACGACGGCCAGT		-
	M13Rev**	CAGGAAACAGCTATGACC		
	ITS2**	GCTGCGTTCTTCATCGATGC		(Lemaire *et al.*, 2011)
	ITS3**	GCATCGATGAAGAACGCAGC		
	ITS_PK1_F1	AACCAAACGACGAGCACCAC		This study
	ITS_PK1_F2	CAACACAGCTGGAGGAAGGAAG		
	ITS_PK1_F3	TAGCAATGCCGCCGAAACTG		
	ITS_PK1_F4	ATCAACGTCCAACAATCAAATG		
	ITS_PK1_F5	CAGCCACAACCAAAACTCTTCTCC		
	ITS_PK1_R1	ACCGTCCTCAAACCCACCTC		
	ITS_PK1_R2	TGAGTCACCGCTGCTTGTTGC		
	ITS_PK2_F1	CAGCAGCAGCAGCAGGAGTG		
	ITS_PK2_F2	CCAACCATCCATACATCCACAAC		
	ITS_PK2_F3	CCAGCCTGCTCACGACCATC		
	ITS_PK2_R1	CGCTCGCTCGCTCGCCTTAC		

[a] Primers were used for both amplification and sequencing (*) or for sequencing only (**); Degenerate nucleotides: y, C or T; h, A, C, or T; r, A or G; w, A or T.

Supplementary Table 3. Pairwise identity matrix of the partial *CYTB* gene sequences of members of the *Prototheca* genus, including three new species.

Species	*P. ciferrii*	*P. zopfii*	*P. cerasi*	*P. bovis*	*P. cookei*	*P. blaschkeae*	*P. cutis*	*P. paracutis*	*P. miyajii*	*P. wickerhamii*	*P. stagnora*	*P. xanthoriae*	*P. moriformis*	*P. tumulicola*	*P. pringsheimii*	*Auxenochlorella* sp.	*Chlorella* sp.	*Chlamydomonas* sp.	*Helicosporidium* sp.	*P. lentecrescens* (PK1/PK6)	*P. fontanea* (PK2)	*P. vistulensis* (W3)	*Prototheca* spp.
P. ciferrii	97.7-100	96.1-97.3	95.7-96.1	95.7-96.9	95.2-96.0	93.2-94.4	85.3-85.7	85.7-86.6	84.4-85.8	86.7-88.1	91.3-91.9	87.0-88.3	87.8-89.3	92.5-93.1	96.8-97.4	86.5-87.1	83.5-85.7	69.5-73.1	84.1-85.5	86.1-87.4	87.6-89.4	93.8-94.4	84.4-97.4
P. zopfii	96.1-97.3	98.5-100	95.4-96.1	96.8-97.1	95.3-96.0	94.1-94.4	85.8-86.2	86.0-87.2	86.1-86.4	87.2-87.9	91.9-92.1	87.9-89.0	88.6-89.7	92.8-93.2	96.5-97.2	87.3-88.2	84.0-85.8	68.4-72.6	85.1-85.3	86.8-87.2	88.8-90.0	94.0-94.7	85.8-97.3
P. cerasi	95.7-96.1	95.4-96.1	99.9-100	95.7-96.0	95.0-96.0	93.5-93.7	86.2-86.4	86.6-87.3	85.6-85.8	87.2-87.8	91.8-91.9	87.0-87.1	88.6-89.7	92.8-92.9	96.8-96.9	86.9-87.1	84.1-86.0	70.4-72.8	85.3-85.5	86.2-86.5	88.1-88.3	94.2-94.4	85.6-96.9
P. bovis	95.7-96.9	96.8-97.1	95.7-96.0	99.9-100	95.7-96.6	93.6-94.0	87.1-87.3	86.5-87.6	86.7-86.9	87.5-88.0	91.6-91.8	88.1-88.2	88.4-89.5	92.7-92.8	96.3-96.4	87.1-87.3	84.5-84.9	69.1-72.2	85.7-86.0	86.6-86.9	88.3-88.5	95.3-95.4	86.5-97.1
P. cookei	95.2-96.0	95.3-96.0	95.0-96.0	95.7-96.6	96.4-100	92.3-93.7	85.9	85.9-86.9	85.9	86.5-87.3	91.4-92.2	87.1-88.0	88.0-90.2	92.3-93.4	95.8-96.0	87.0-87.2	83.8-85.9	70.3-72.7	85.2-85.7	86.5-87.0	88.2-88.6	94.4-94.6	85.9-96.6
P. blaschkeae	93.2-94.4	94.1-94.4	93.5-93.7	93.6-94.0	92.3-93.7	100	85.3-85.7	85.8-86.5	86.2-86.3	86.2-87.9	90.3-90.4	86.8-87.1	87.3-88.5	91.4-91.5	94.4	86.7-86.8	83.1-85.9	69.3-72.8	86.1	88.2-88.4	88.9-89.0	92.4-92.7	85.3-94.4
P. cutis	85.3-85.7	85.8-86.2	86.2-86.4	87.1-87.3	85.9	85.3-85.7	100	97.0-98.3	91.0	89.7	84.4	90.1	84.8-85.3	85.4	86.1	89.4	82.4-85.5	67.4-71.4	86.0	88.6-88.7	87.6	85.2	84.4-98.3
P. paracutis	85.7-86.6	86.0-87.2	86.6-87.3	86.5-87.6	85.9-86.9	85.8-86.5	97.0-98.3	98.9-100	90.6-91.8	89.2-90.2	84.7-85.8	88.8-90.0	84.2-85.5	86.0-86.8	86.5-87.1	88.7-90.0	82.3-86.7	66.7-70.6	84.6-85.6	88.6-89.6	87.9-88.7	85.6-85.7	84.2-98.3
P. miyajii	84.4-85.8	86.1-86.4	85.6-85.8	86.7-86.9	85.9	86.2-86.3	91.0	90.6-91.8	100	89.5-90.1	86.7	90.5	84.8-85.3	87.1	87.1	89.0	82.8-84.7	67.1-70.5	86.0	89.4-89.5	90.2	84.5	84.4-91.8
P. wickerhamii	86.7-88.1	87.2-87.9	87.2-87.8	87.5-88.0	86.5-87.3	86.2-87.9	89.7	89.2-90.2	89.5-90.1	99.4-100	86.0-86.5	90.2-90.8	84.2-85.1	87.4-87.9	88.0-88.2	88.8-89.2	83.8-85.4	68.3-72.5	86.0-86.1	90.6-91.5	89.8-90.3	85.2-85.5	84.2-91.5
P. stagnora	91.3-91.9	91.9-92.1	91.8-91.9	88.1-88.2	91.4-92.2	90.3-90.4	84.4	84.7-85.8	86.7	86.0-86.5	100	87.8	89.2-90.0	93.8	92.1	87.2	82.6-84.1	69.5-73.4	83.8	85.9-86.1	86.9	89.5	84.4-93.8
P. xanthoriae	87.0-88.3	87.9-89.0	87.0-87.1	88.4-89.5	87.1-88.0	86.8-87.1	90.1	88.8-90.0	90.5	90.2-90.8	87.8	100	85.1-85.8	87.3	87.9	93.0	85.2-87.6	66.5-70.6	87.6	90.9-91.0	91.8	86.1	85.1-91.8
P. moriformis	88.6-89.7	85.7-87.1	88.6-89.7	88.4-89.5	88.0-90.2	87.3-88.5	84.8-85.3	84.2-85.5	84.8-85.3	84.2-85.1	89.2-90.0	85.1-85.8	98.2-100	90.6-91.3	89.0-89.5	84.7-85.1	82.6-84.7	68.1-71.5	83.0-83.2	84.5-85.6	85.1-85.7	86.9-87.8	84.2-91.3
P. tumulicola	92.5-93.1	92.8-93.2	92.8-92.9	92.7-92.8	92.3-93.4	91.4-91.5	85.4	86.0-86.8	87.1	87.4-87.9	93.8	87.3	90.6-91.3	100	94.1	87.5	84.9-86.9	69.4-73.3	84.1	86.7-86.9	89.2	90.7	85.4-94.1
P. pringsheimii	96.8-97.4	96.5-97.2	96.8-96.9	96.3-96.4	95.8-96.0	94.4	86.1	86.5-87.1	87.1	88.0-88.2	92.1	87.9	89.0-89.5	94.1	100	87.7	84.5-86.5	70.3-73.1	86.3	87.2-87.3	88.9	94.2	86.1-97.4
***Auxenochlorella* sp.**	86.5-87.1	87.3-88.2	86.9-87.1	87.1-87.3	87.0-87.2	86.7-86.8	89.4	88.7-90.0	89.0	88.8-89.2	87.2	93.0	84.7-85.1	87.5	87.7	100	83.6-86.3	66.2-71.6	87.0	88.4-88.5	89.3	86.0	84.7-93.0
***Chlorella* sp.**	83.5-85.7	84.0-85.8	84.1-86.0	84.5-84.9	83.8-85.9	83.1-85.9	82.4-85.5	82.3-86.7	82.8-84.7	83.8-85.4	82.6-84.1	85.2-87.6	82.6-84.7	84.9-86.9	84.5-86.5	83.6-86.3	89.3-100	66.2-70.3	81.5-82.9	85.1-86.7	84.5-86.3	81.7-83.9	81.7-87.6
***Chlamydomonas* sp.**	69.5-73.1	68.4-72.6	70.4-72.8	69.1-72.2	70.3-72.7	69.3-72.8	67.4-71.4	66.7-70.6	67.1-70.5	68.3-72.5	69.5-73.4	66.5-70.6	68.1-71.5	69.4-73.3	70.3-73.1	66.2-71.6	66.2-70.3	81.7-100	67.4-70.4	67.7-70.4	68.3-71.7	70.2-71.8	66.5-73.4
***Helicosporidium* sp.**	84.1-85.5	85.1-85.3	85.3-85.5	85.7-86.0	85.2-85.7	86.1	86.0	84.6-85.6	86.0	86.0-86.1	83.8	87.6	83.0-83.2	84.1	86.3	87.0	81.5-82.9	67.4-70.4	100	86.6-86.7	84.7	84.4	83.0-87.6
***P. lentecrescens* (PK1/6)**	86.1-87.4	86.8-87.2	86.2-86.5	86.6-86.9	86.5-87.0	88.2-88.4	88.6-88.7	88.6-89.6	89.4-89.5	90.6-91.5	85.9-86.1	90.9-91.0	84.5-85.6	86.7-86.9	87.2-87.3	88.4-88.5	85.1-86.7	67.7-70.4	86.6-86.7	99.9-100	90.6-90.7	85.1-85.3	84.5-91.5
***P. fontanea* (PK2)**	87.6-89.4	88.8-90.0	88.1-88.3	88.3-88.5	88.2-88.6	88.9-89.0	87.6	87.9-88.7	90.2	89.8-90.3	86.9	91.8	85.1-85.7	89.2	88.9	89.3	84.5-86.3	68.3-71.7	84.7	90.6-90.7	100	86.5	85.1-91.8
***P. vistulensis* (W3)**	93.8-94.4	94.0-94.7	94.2-94.4	95.3-95.4	94.4-94.6	92.4-92.7	85.2	85.6-85.7	84.5	85.2-85.5	89.5	86.1	86.9-87.8	90.7	94.2	86.0	81.7-83.9	70.2-71.8	84.4	85.1-85.3	86.5	100	84.5-95.4
